# Gene Therapy Advances: A Meta-Analysis of AAV Usage in Clinical Settings

**DOI:** 10.3389/fmed.2021.809118

**Published:** 2022-02-09

**Authors:** Hau Kiu Edna Au, Mark Isalan, Michal Mielcarek

**Affiliations:** ^1^Department of Life Sciences, Imperial College London, London, United Kingdom; ^2^Imperial College Centre for Synthetic Biology, Imperial College London, London, United Kingdom

**Keywords:** adeno-associated virus, gene therapy, tropism, clinical trials, promoters

## Abstract

Adeno-associated viruses (AAVs) are the safest and most effective gene delivery vehicles to drive long-term transgene expression in gene therapy. While animal studies have shown promising results, the translatability of AAVs into clinical settings has been partly limited due to their restricted gene packaging capacities, off-target transduction, and immunogenicity. In this study, we analysed over two decades of AAV applications, in 136 clinical trials. This meta-analysis aims to provide an up-to-date overview of the use and successes of AAVs in clinical trials, while evaluating the approaches used to address the above challenges. First, this study reveals that the speed of novel AAV development has varied between therapeutic areas, with particular room for improvement in Central Nervous System disorders, where development has been slow. Second, the lack of dose-dependent toxicity and efficacy data indicates that optimal dosing regimes remain elusive. Third, more clinical data on the effectiveness of various immune-modulation strategies and gene editing approaches are required to direct future research and to accelerate the translation of AAV-mediated gene therapy into human applications.

## Introduction

Gene therapy functions by introducing genetic materials into patients to alter gene or protein expression, potentially providing a one-time curative treatment for many diseases that currently have no cure (1, 2). Four decades of research has shown that adeno-associated viruses (AAVs) appear to be the safest and most effective delivery vehicles, or vectors, to deliver genes of interest into a broad range of cell types in gene therapy. Hereditary diseases are particularly attractive targets; these are caused by gene mutations, resulting in deficiency or malfunction of proteins required for cellular functions. To treat such hereditary diseases at their source, gene therapy can correct disease mutations in three ways—(1) to replace the defective gene with a functional copy, (2) to silence the mutated version of the gene, and (3) to add or overexpress a therapeutic gene or synthetic construct. Silencing can be achieved by introducing a short hairpin RNA, embedded into a microRNA structure (3), or by zinc finger silencing technology (4, 5). All of the above can be done either transiently, by delivering a gene as a DNA episome which remains physically independent of the cell's chromosome but is stable in the nucleus, or permanently, by editing the genome using specific techniques such as zinc finger nucleases (ZFNs) (6). Both transient and permanent approaches can be mediated by AAVs.

**Figure 1:**
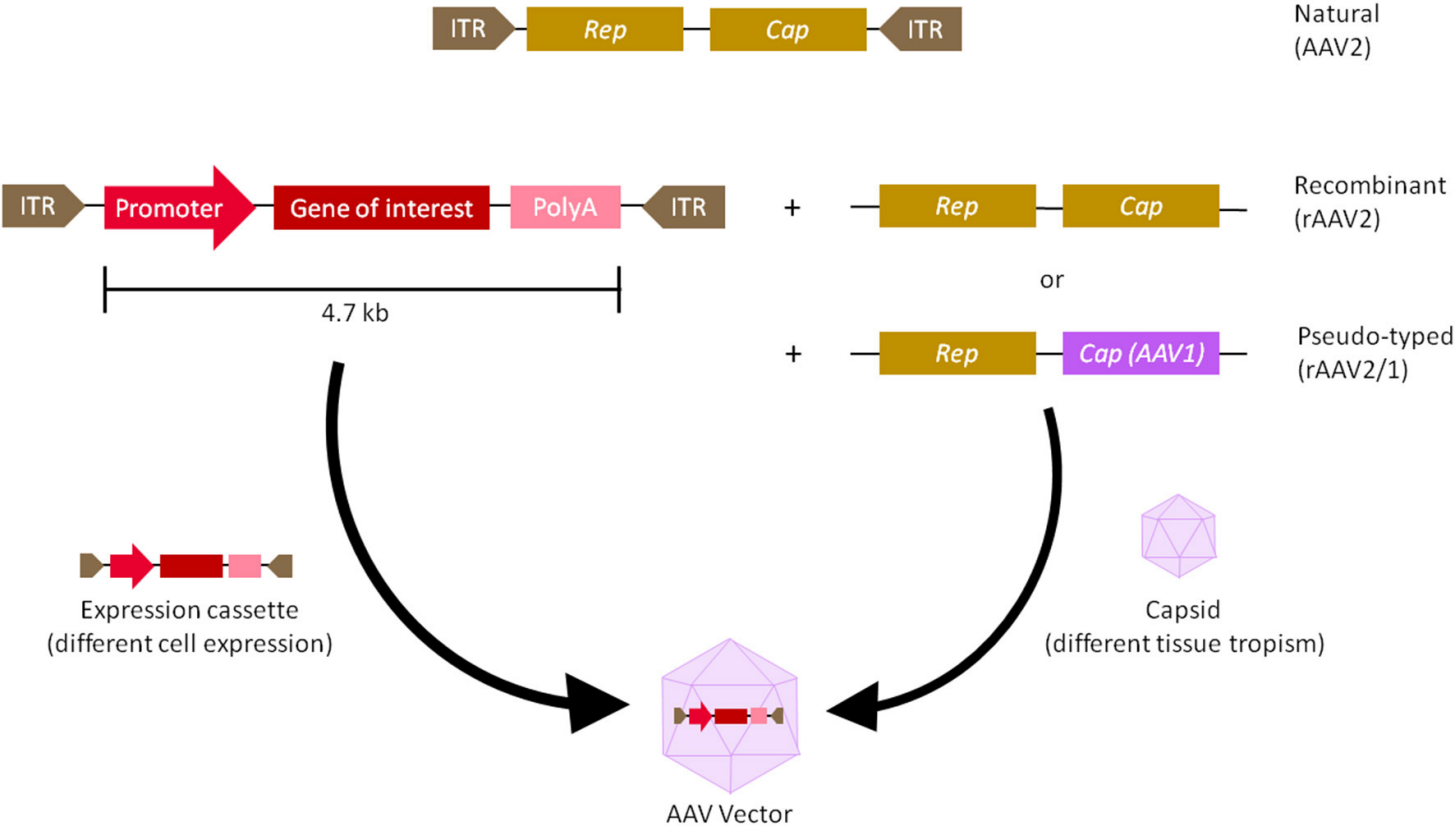
Adeno-associated virus (AAV) vector biology. The natural AAV serotype contains three open reading frames flanked by two inverted terminal repeats (ITR)—*Cap* (capsid) and *Rep* (replication). In recombinant AAVs (rAAV), the viral genome is replaced by a synthetic expression cassette containing a promoter, transgene of interest and a terminator such as polyadenylation (polyA) sequence, flanked by the ITRs. *Cap* and *rep* sequences are supplied as a plasmid *in trans*, which will produce the viral capsid that packages the expression cassette. To create a pseudo-typed vector (e.g., AAV2/1), the *cap* genes from another AAV serotype (e.g., AAV1) can be used to package the recombinant genome of another serotype (e.g., AAV2). rAAVs can be customised at the capsid and promoter level. The capsid gives AAV its tissue tropism, meaning the cells it infects, while the promoter drives either ubiquitous or tissue-specific expression of the transgene.

AAVs are small, non-enveloped viruses with a single-stranded genome DNA of 4.7 kb, flanked by 2 Inverted Terminal Repeats (ITRs). There are 3 genes within the viral genome—*Rep* (Replication) is responsible for viral replication and packaging, *Cap* (Capsid) encodes 60 outer coat proteins that protect the genomic DNA and direct cell binding, and *Aap* (Assembly activating protein) provides a scaffold for capsid assembly (7, 8).Over the past couple of decades, recombinant AAVs (rAAVs) were engineered to have most of the viral genomes replaced with expression cassettes containing a promoter, genes of interest, and a terminator, in order to make them more suitable for clinical applications (Figure 1). As such AAVs cannot replicate, they are a very safe vehicle to drive long-term transgene expression after a single infection (1, 9). In fact, the longest AAV transgene expression reported lasted over 15 years in primates (10). AAVs' simple genomes also make them very versatile and ideal for engineering. However, AAV vectors package single-stranded genomes and require host-cell synthesis of the complementary strand for transduction and this event is one of the rate-limiting steps for transgene expression (11). Briefly, the single-stranded (ss) DNA molecule with a length of ~4.7 kb can exist either in plus- or in minus-form, which requires a conversion into double-stranded (ds) DNA either by strand annealing of one plus- and one minus-strand or by ”*de novo*” synthesis of DNA prior to gene expression (12, 13). In order to overcome this problem, self-complementary (sc) AAV vectors have been introduced as these vectors contain a dimeric inverted repeat genome that allows folding into dsDNA (11, 14). However, this approach has a major disadvantage as scAAV vectors have even more limited coding capacity in comparison to ssAAV. This could be an obstacle in delivering large constructs containing full length genes.

Moreover, the promoters can be viral or native to the host organism, driving either ubiquitous or tissue-specific expression. Other enhancers, introns, polyadenylation sequences, transcript stabilising elements and codon host optimization may be added as well to tune transgene expression (4, 15).

Chosen expression cassettes are packaged into different AAV capsids with different tissue tropisms (16). Traditional capsids are isolated from natural sources, usually from humans and primates. Out of the main 11 serotypes (AAV1 to AAV11) cloned thus far, historically, AAV2 is the best characterised and is generally thought to have suitably safe and efficient packaging and transduction capabilities. It is often used as the backbone for pseudotyped vectors—a hybrid vector, in which ITRs of one serotype are packaged in the capsid of another serotype, to alter its tissue tropism (Figure 1) (17). Promising new variants have also been isolated from rhesus macaque, such as AAVrh.10 and AAVrh.74, with good transduction properties and lower pre-existing human population immunity against them, compared to AAV2 (2, 18, 19). More recently, novel vectors were created by rational design, in which site-specific modifications were made to the capsid in order to improve tissue specificity or modify antigenic sites (20). Variants with improved gene delivery properties have also been identified by directed evolution, in which a diverse library of capsids was generated by gene shuffling and random mutagenesis, followed by a selection of the fittest (7). Selecting the right capsid and promoter is the first step to improve targeted cell transduction and expression (2, 15). This has important implications for a second step—establishing dosing regime. Dosages that are too low could lead to inefficient transduction, whereas dosages that are too high can result in delivery and transduction-related toxicities (21). Moreover, for transgenes that can also correct neighbouring cells via so-called “by-stander effects,” maximising cell transduction may not be necessary (22, 23). Lower dosage is also beneficial from the manufacturing point of view, since the current production capacity of GMP grade AAVs is typically capped at ~5 × 10^13^ viral particles/ml concentration (7). Therefore, the minimal dosage required to reach therapeutic significance would be the safest and most ideal.

Lastly, and perhaps the biggest hurdle that needs addressing for human applications, is the development of immune responses against the viral capsid and delivered transgene, especially when the vector is being systemically administered (15, 24). Most people have already been exposed to wild-type AAVs and thus have pre-existing adaptive immunity, including neutralising antibodies (NAbs) and T cells against some AAVs, potentially leading to loss of transgene expression or elimination of transduced cells. This heavily undermines clinical efficacy and prevents re-administration (7, 25). The known prevalence of pre-existing anti-capsid NAbs in the population varies for different AAV variants, ranging from 40% for anti-AAV8 to 74% for anti-AAV2 NAbs. Co-prevalence and cross-reactivity of NAbs against other serotypes further complicates matters, by preventing simply switching gene therapy to a different serotype (26).

**Figure 2:**
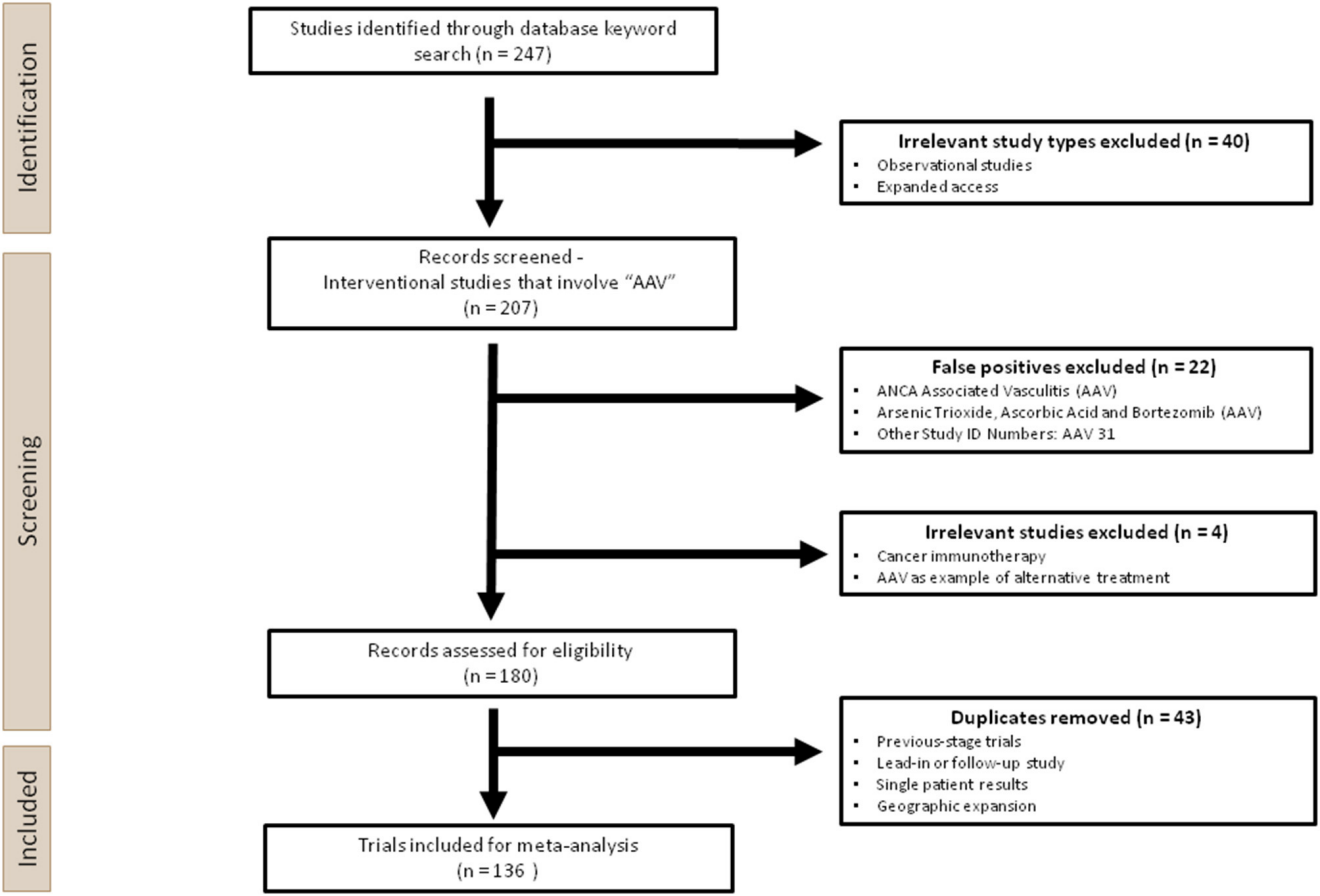
Overview of the experimental design used in this analysis. Two hundred forty-seven clinical trials were first identified through a keyword search for “AAV” on ClinicalTrials.gov. Two hundred seven interventional studies were screened, during which irrelevant studies, false positives and duplicates were removed manually. One hundred thirty-six trials were ultimately included for the meta-analysis.

By analysing the 136 trials identified in this meta-analysis, we aim to understand the current landscape and provide an up-to-date overview of the use of AAVs in clinical trials (Figure 2). Our analysis will focus on the three challenges: choice of AAV construct (promoter and capsid), dosing regime and AAV-mediated immunogenicity. Our analysis will summarise current trends and gaps in order to accelerate the translation of AAV-mediated gene therapy into human applications.

## Materials and Methods

This systematic analysis was performed according to the 2020 PRISMA guidance, which is used for reporting meta-analyses on health interventions (27). A total of 247 trials were extracted from the U.S National Library of Medicine database (ClinicalTrials.gov), the largest clinical trials database to date, following the keyword search for “AAV,” using the cut-off of 26 April 2021. Prior to screening, Observational Studies and Expanded Access were removed since they would not involve AAV administration. During the screening process, 22 studies were excluded as false positives, which contained the search term “AAV” as a short-hand for terms other than adeno-associated virus. For those that used “AAV” appropriately, three trials were excluded since AAV was not the main intervention product, or the application was not gene therapy, hence these are out of the scope of this analysis. One hundred thirty-six unique trials were identified after removing 44 duplicates (Figure 2).

Since most information was not fully available on the registry, published results, and research papers including previous pre-clinical studies using the same vector were reviewed. If the vector construct and study design were not sufficiently described in published papers, the search was broadened to (1) company registration documents (SEC and IPO filings), (2) patents, (3) company's website, (4) company's presentations and/or press releases, and (5) results published in international research conferences.

Information on the study design relevant to this analysis is as follows: delivery method, target tissue and co-delivered drugs, if any. Exclusion criteria relevant to anti-capsid antibodies and immunosuppression were also summarised. Missing information was marked as “N/A.” The data can be found in the Supplementary Table 1.

## Results

**Figure 3:**
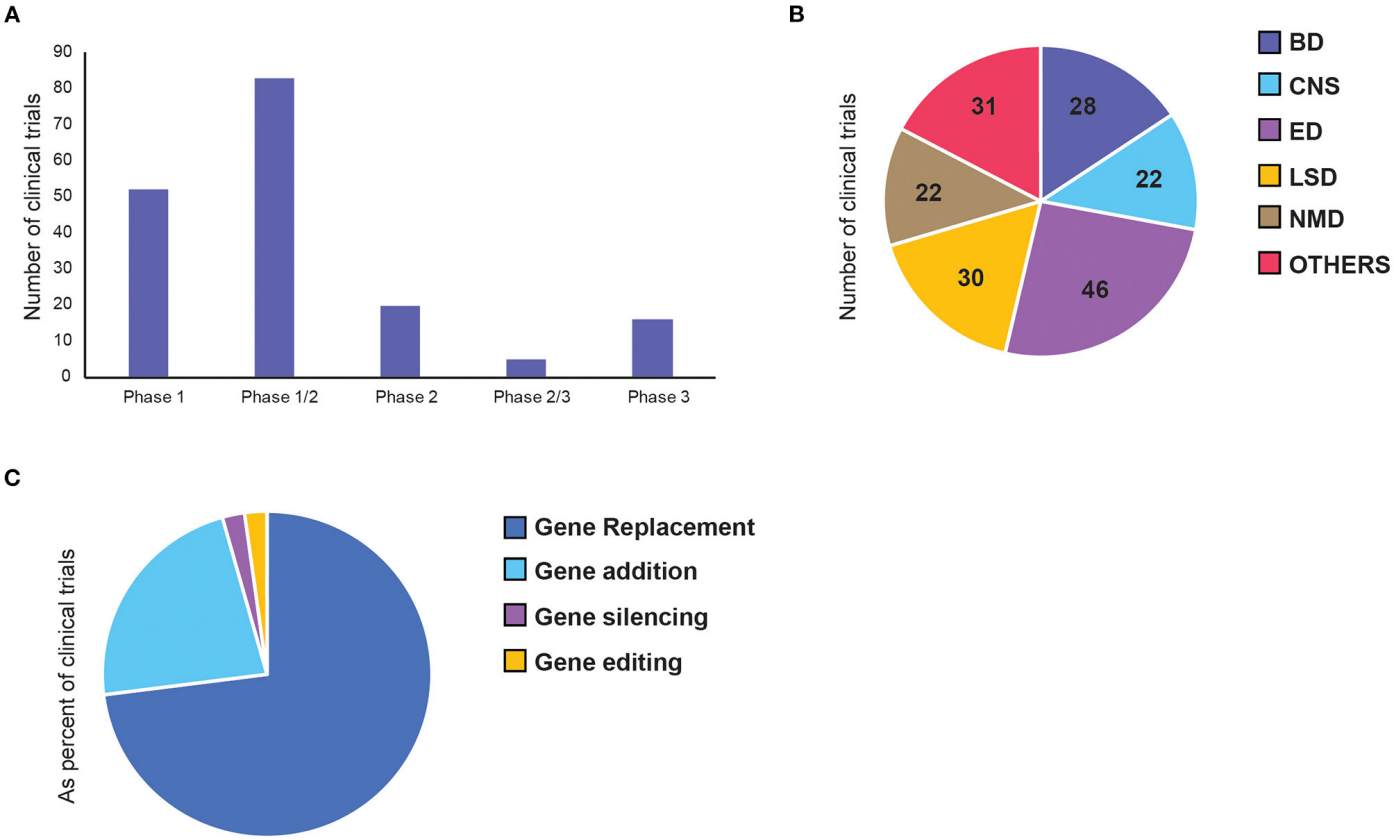
Distribution of adeno-associated virus (AAV)-mediated gene therapy in clinical trials. Each panel represents number of clinical trials by **(A)** current status of the clinical phase, **(B)** therapeutic area, and **(C)** gene therapy approaches. BD, Blood disorders; CNS, Central Nervous System; ED, Eye Disorders; LSD, Lysosomal storage disorders; NMD, Neuromuscular Disorders.

Overall, we identified 136 clinical trials involving 134 AAV drug products to treat 55 diseases (Supplementary Table 1). Majority of clinical trials used single stranded AAVs, while only 7 used self-complementary (sc) AAV vectors (see Extended Supplementary Material). Since most of the therapeutic targets are rare diseases, 64% of the trials were combined phases (phase 1/2 or phase 2/3). However, 75% of all trials are still at early-phase (phase 1 or phase 1/2) with low translation rate into phase 2 or 3 (Figure 3A). Of the 23 late-stage trials, only two received FDA approval for commercialisation so far—Luxturna for retinal dystrophy (NCT00999609) and Zolgensma for spinal muscular atrophy (SMA) (NCT03306277).

However, the first approved human gene therapy based on AAV delivery was Glybera (alipogene tiparvovec) that received marketing authorisation by the European Medicines Agency in 2012 (28). This drug was designed for a treatment of the inherited metabolic disorder lipoprotein lipase (LPL) deficiency (LPLD) (29), but unfortunately the product was withdrawn from the market in 2017.

The majority of the trials can be categorised into five broad therapeutic areas—blood disorders (BD), central nervous system (CNS) disorders, eye disorders (ED), lysosomal storage disorders (LSD), and neuromuscular disorders (NMD). Less represented diseases were grouped as “Others,” which include diseases affecting: the heart, lung, liver, as well-inflammatory, HIV and cancer (Figure 3B). Inherited eye disorders cause retinal degeneration that can lead to progressive photoreceptor cell death and vision loss. This area makes up the largest proportion (34%) of the clinical landscape with 30 trials, all involving single targeted delivery of AAV vectors to the retina. Twenty percentage of these are pivotal phase 3 or 2/3 trials, making it the most advanced across all disease groups. Lysosomal storage disorders are a group of rare metabolic diseases caused by inherited deficiency in lysosomal enzymes, resulting in accumulation of excess substrates in various organs (30). This is the second largest therapeutic area within AAV gene therapy, mainly made up of replacement therapy for intracellular enzymes. Blood disorders in AAV gene therapy consists of two main bleeding disorders, namely haemophilia A and B, resulting from deficiency in coagulation factor VIII (FVIII) and IX (FIX), respectively (24). All trials for BDs were made up of single-dose, intravenous (IV) administration of a functional gene replacement for hepatocyte expression. Central nervous system (CNS) disorders are particularly difficult to treat due to complex cell networks and the blood-brain barrier (BBB) limiting access to brain structures. Out of the four trials that progressed to phase 2, three have failed, with one awaiting results at the end of 2022. Neuromuscular disorders form the smallest of the key therapeutic areas, made up of a group of inherited and acquired conditions that affect motor neurons and skeletal muscles. Some of these are well-defined monogenic diseases, such as Duchenne muscular dystrophy and spinal muscular atrophy, making them promising targets for gene therapy (31).

Next, we also classified gene therapy approaches based on the characteristics of the core therapeutic functions and divided them into 4 classes: gene replacement, gene addition, gene silencing and gene editing (Figure 3C). Among them, over 70% of the trials are based on gene replacement, and over 20% are gene addition (Figure 3C). There are only three gene silencing and three genome editing trials, four of which were only initiated in the past 5 years, suggesting their research in human applications is still in its infancy. The first gene silencing approach used short hairpin RNAs (shRNAs), which bind the target gene in host cells to inactivate them directly. To treat patients with hepatitis C (HCV) infection, anti-HCV sequences were delivered to cleave the HCV genes. This trial was completed in 2016 and proved to be safe. However, only 1–2% hepatocytes were transduced and there was no efficacy data on whether silencing was achieved (32). Another shRNA was used later to inhibit the transcription of mutant HTT gene, which is an elongated version of the wild-type protein that gets broken down into toxic fragments in Huntington's disease (33). The second gene silencing approach is a splice-site inhibitor, currently being tested to treat Duchenne Muscular Dystrophy, caused by duplication of exon 2 of the dystrophin (DMD) gene. The splice site of exon 2 can be blocked by U7-ACCA, a non-coding U7 small nuclear RNA, causing significant exon skipping so the wild-type (or a highly-functional exon-2-deleted form) can be expressed instead. Both the latter trials are still ongoing, with expected completion in 2022 (NCT04120493) and 2023 (NCT04240314) respectively.

There are three attempts at genome editing so far, all mediated by zinc finger nucleases (ZFN). The first, an attempt to treat Mucopolysaccharidosis type I, was completed in 2013 with no results posted. Meanwhile, the second, for Mucopolysaccharidosis type II, awaits results at the end of 2021. The most recent trial delivering SB-FIX to treat haemophilia B was recently terminated on May 3, 2021, after enrolling the first patient, for an unknown reason (NCT02695160). To date, there appear to be no positive results on the clinical efficacy of such gene therapies, in either silencing or editing approaches.

### Vector Construct—Capsid Type and Promoter Choice

A total of 17 different AAV capsids and 27 promoters were disclosed in clinical trials, with their distribution across the therapeutic areas summarised in the Supplementary Table 1.

**Figure 4:**
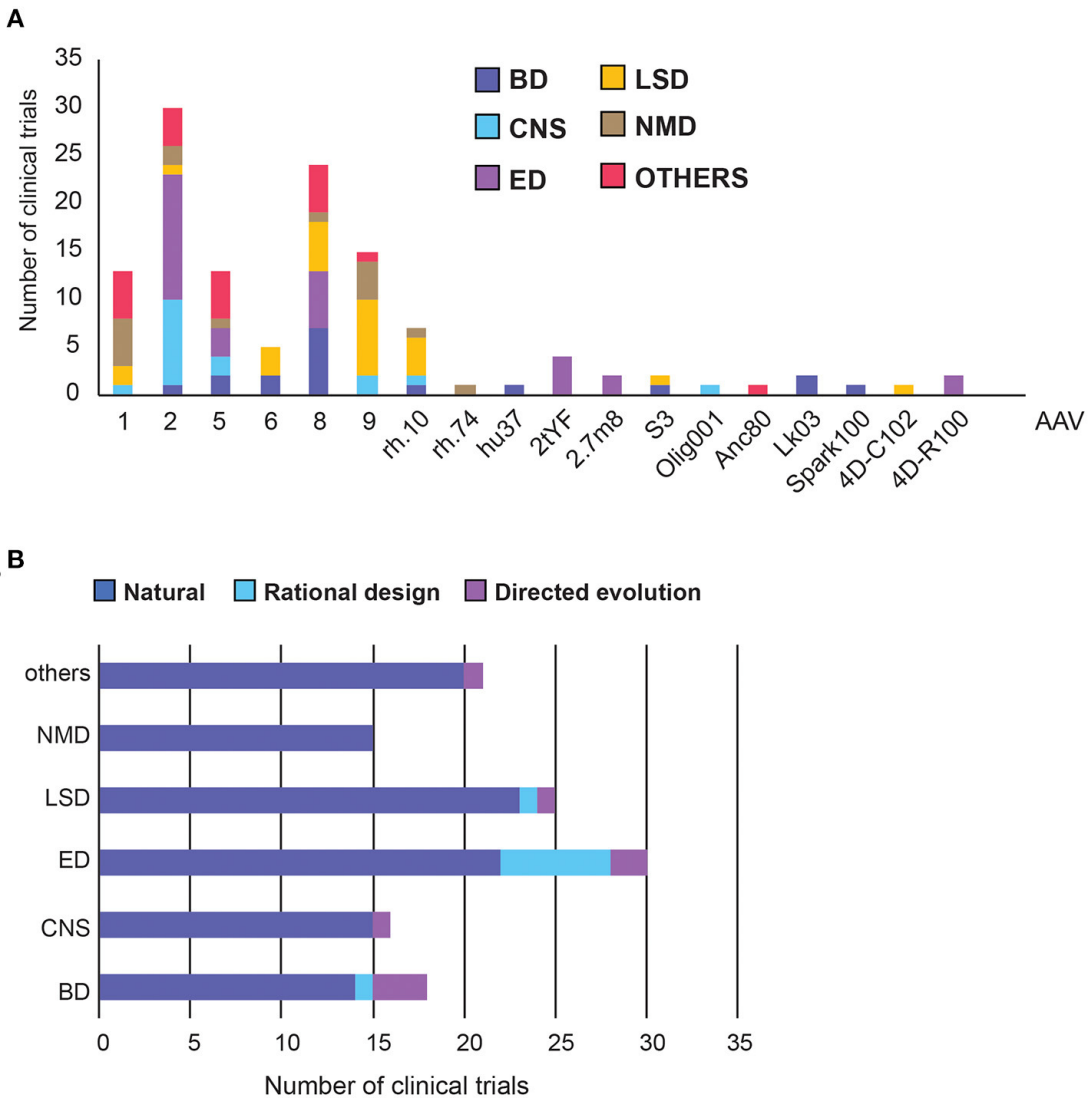
Adeno Associated Virus (AAV) capsid usage and frequency in clinical trials. **(A)** Overall AAV capsid type usage across all clinical trials. **(B)** Capsid design across therapeutic areas. BD, Blood disorders; CNS, Central Nervous System; ED, Eye Disorders; LSD, Lysosomal storage disorders; NMD, Neuromuscular Disorders. The list of capsid descriptions can be found in Supplementary Table 2, Supplementary Figure 1.

Out of the 125 disclosed AAV capsids, 87% were from natural serotypes or were pseudo-typed, meaning the transgene, flanked by AAV2 ITRs, was packaged in the capsid of another serotype (Figure 4A, Supplementary Figure 1) (17). We further classified capsids into three categories: natural, rational design, or directed evolution (Figure 4B). Full descriptions of each AAV capsid can be found in Supplementary Table 2.

AAV2 is the most commonly used serotype, making up 30% of all capsids and was found predominantly in the treatment of Eye and CNS disorders (Figure 4A). This is perhaps unsurprising as AAV2 was the first serotype to be cloned, and is currently patent-free (34). Notably, each disease group appears to have a capsid preference, likely due to the different tissue tropism each serotype exhibits. AAV8 has been used the most frequently in Blood Disorders, AAV9 in LSDs, AAV1 and 9 in neuromuscular disorders, and a relatively even distribution in other disorders (Figure 4A). Across therapeutic areas, Eye Disorders had the highest proportion of use of novel capsids (27%), followed by Blood Disorders (22%). The rest are lagging behind considerably, from none in Neuromuscular Disorders to just 8% in LSDs (Figure 4A). In Blood Disorders, AAV8 is being used the most frequently due to its tropism for the liver; it appears in almost 40% of the trials. This is likely due to proven success of a scAAV8-co*F9* vector, providing stable expression of FIX protein for over 11 years in haemophilia B patients (35, 36). There are only two novel capsids used in Blood Disorders, namely LK03 and Spark100. LK03 is the first novel capsid produced by capsid shuffling and library selection, and is being used in two trials to treat haemophilia A. It has a *cap* sequence made up of fragments from seven different wild-type serotypes (AAV1, 2, 3B, 4, 6, 8, 9), and was shown to transduce human hepatocytes 100-fold better than AAV8 *in vitro*, along with being resistant to NAbs (37). Spark100 is another engineered capsid currently being prepared for phase 3 clinical trials, after demonstrating sustained therapeutic expression of FIX coagulant activity following gene transfer to Haemophilia B patients (38). It should be noted that Spark100 is only 12 amino acids different from the AAV8 capsid (39).

Vectors targeting CNS used the “least-novel” capsids, with all but one capsid based on natural serotypes. AAV2 makes up over half of the vectors, likely due to its established neuronal tropism (40). However, AAVrh.10 showed a greater transgene enzyme distribution within the brain and a better immunogenicity profile than rAAV2 in pre-clinical studies, and has therefore been used in two clinical trials to treat Alzheimer's Disease (NCT03634007) and Batten Disease (NCT01161576) (8). Nonetheless, while trial results showed good safety profiles, they did not slow disease progression to the extent of the current standard-of-care replacement therapy for Batten Disease (41). The only novel vector used in CNS trials is AAV/Olig001, which contains a chimeric mixture of AAV1, 2, 6, 8, and 9, generated using capsid shuffling and directed evolution. It is the first AAV vector that exhibits strong striatal and oligodendroglial tropism without the need for cell-specific promoters (42).

Despite having the highest number of trials, only six different capsid types were used in Eye Disorders. More than half of them are the natural AAV2 capsid, from which two novel capsids are derived (43). In the AAV2tYF variant, multiple surface tyrosine residues were mutated to phenylalanine to prevent tyrosine phosphorylation, which could trigger ubiquitination and proteasome-mediated degradation of the AAV. AAV2tYF was shown to drive stronger and more widespread transgene expression in retinal cells compared with their wild-type counterparts, and was used by three different companies in clinical trials (44). AAV2.7m8 is an engineered capsid with a 10-amino acid insertion in the surface variable region VIII of the capsid, in an attempt to alter the antigenic region of AAV2 and avoid immune activation. It was also shown to transduce retina cells efficiently (45). 4D-R100 is 4D Molecular Therapeutics' proprietary vector optimised through directed evolution to efficiently transduce all layers of the retina. It is being used in two clinical trials to treat Choroideremia and Retinitis Pigmentosa (46).

For LSDs, the natural serotypes AAV9, AAV8, and AAVrh.10 are the most commonly used capsids, along with two novel capsids. The first, AAVS3, is a rationally-designed capsid with exceptional efficiency in transducing hepatocytes, achieved by swapping gene sequences demonstrating human liver tropism into the domains of native serotypes (47). The second, 4D-C102, is a novel muscle-specific variant, identified through directed evolution, and showing superior gene delivery and reduced immunogenicity over AAV1, 8 and 9 in cardiac and liver cells in mice (48). It is currently being trialled for the treatment of Fabry disease in humans (NCT04519749).

The vector usage in neuromuscular and other disorders is even more conservative, with only one novel vector, Anc80, used to treat Wilson's disease in the liver. Anc80 is produced by backwards-directed evolution, which computationally predicts and experimentally recreates ancestors of contemporary AAV capsids. Anc80 represents the ancestor of AAV1, 2, 8, and 9 (49).

**Figure 5:**
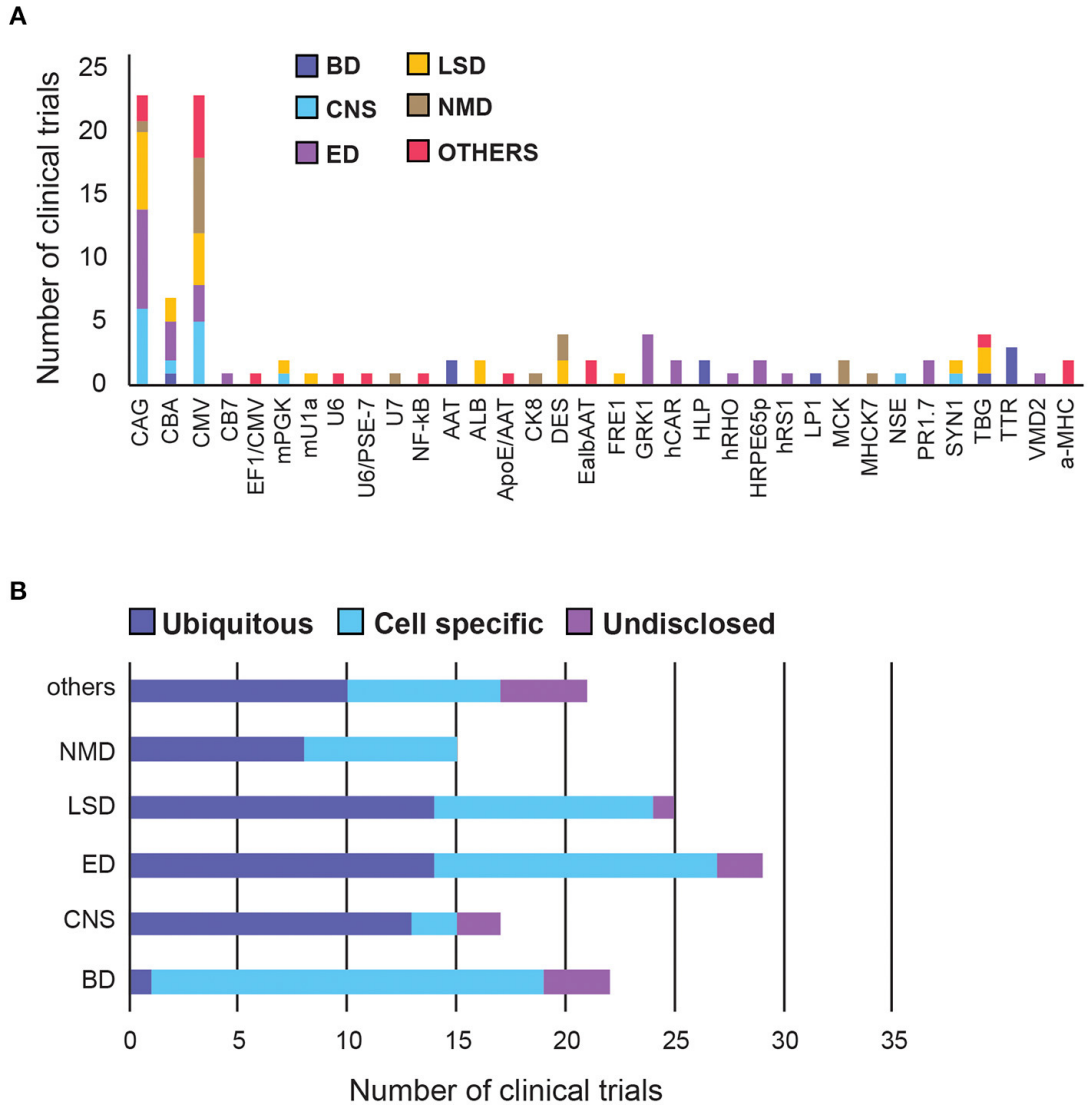
A summary of promoter choice for AAV gene therapy delivery in clinical trials. **(A)** Overall promoter distribution in all clinical trials. **(B)** Promoter type across therapeutic areas. BD, Blood disorders; CNS, Central Nervous System; ED, Eye Disorders; LSD, Lysosomal storage disorders; NMD, Neuromuscular Disorders. The list of promoter abbreviations can be found in Supplementary Table 3, Supplementary Figure 2.

We next researched the choice of the promoter in clinical trials. Out of the 106 disclosed promoters, over 50% are one of: CBA (chicken beta-actin), CMV (cytomegalovirus) or CAG (a synthetic promoter consisting of CMV enhancer, CBA promoter and a rabbit beta-globin splice acceptor). This restricted variety is likely because these are strong ubiquitous promoters with demonstrated track records of efficiency. CAG has been used the most in LSD and Eye Disorders, whereas CMV promoter was used in Neuromuscular and Other diseases (Figures 5A,B). The usage of ubiquitous and tissue-specific promoters varies across therapeutic areas. Seventy-six percentage of promoters used for CNS disorders are ubiquitous, vs. only 5% in blood disorders. The rest are fairly evenly distributed across other therapeutic areas (Figure 5B, Supplementary Figure 2).

Blood Disorders have the lowest diversity of promoters but the highest proportion (95%) of tissue-specific promoters among all therapeutic areas, perhaps to circumvent the generality of the systemic administration route.

In CNS there are two leading ubiquitous promoters being frequently used in clinical settings, namely CMV and CAG. Interestingly, two other promoters, mPGK and hSYN, have been shown to direct stronger transgene expression in the brain and spinal cord, compared to CAG and CMV (50), however their translation into the clinic has been slow. Similarly, the first neurone-specific NSE promoter was used in clinical trials to deliver GAD (glutamic acid decarboxylase) in Parkinson's patients in 2005, but has taken over 15 years to move into phase 2, in early 2021 (51, 52).

Eye Disorders have the highest promoter diversity, with half of them being tissue-specific, targeting photoreceptors, or retinal pigment epithelium (RPE). The 1.7-kb L-opsin promoter (PR1.7) is a synthetic promoter based on the human red opsin gene enhancer and promoter sequences. It exhibited strong and specific GFP expression in all cone photoreceptors, in preclinical models, and is currently being tested in clinical trials to deliver a functional cyclic nucleotide-gated channel (CNGA3) gene to treat Achromatopsia (53). There is also an hRPE65 promoter optimised by excising the inhibitory elements of the promoter using naturally-occurring restriction enzyme sites, which conferred higher expression and specificity to the RPE *in vivo* (54).

LSDs and Neuromuscular Disorders have an even mix of ubiquitous and tissue-specific promoters, likely because they affect multiple organs. While there is a slight preference for CAG for LSDs, there is a strong preference for CMV in neuromuscular diseases.

Additional novel promoter strategies, such as hybrid and dual promoters, have emerged in recent years, mostly in therapeutic areas other than the major five categories. For example, PSE-7/U6-1 is a hybrid Pol III promoter with hybridised PSE-7 sequences inserted into the U6-1 promoter, which significantly reduce the transcription of a potentially toxic shRNA product during gene silencing (55). Furthermore, CMV and EF1a can be used as dual promoters to simultaneously express the variable heavy and light chains of IgG1 antibody in different cassettes, allowing a larger protein to self-assemble *in vivo* (56).

### AAV Dosing Regime

We also examined the range of dosages used in clinical trials thus far. We divided all trials into two categories: systemic, when AAVs were delivered though intravenous injection, and targeted, when AAVs were injected directly into the affected tissue. Overall, systemically administrated AAV vectors require higher dosages than those delivered to targeted sites.

**Figure 6:**
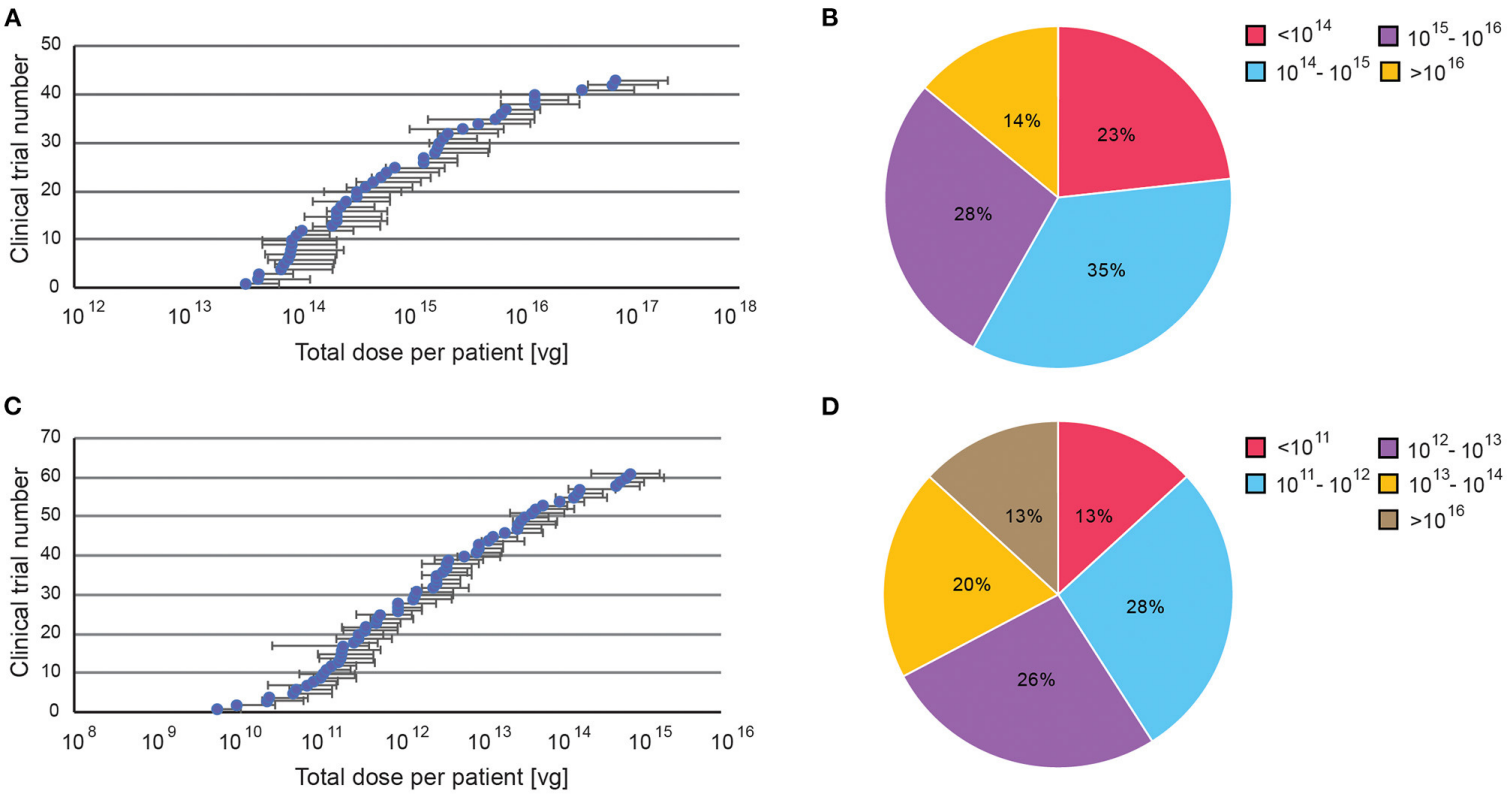
A summary of dosage regime of AAV administered in clinical trials. Dosages were reported in viral genomes (vg) administrated per patient per dose, via either systemic or targeted administration. **(A)** Range of doses in systemic administration, **(B)** Percentage of clinical trials with specific AAV dose windows for systemic administration. **(C)** Range of doses in targeted administration (if disclosed). **(D)** Percentage of clinical trials with specific AAV dose windows for targeted administration. All data presented as a total injected dose per patient. For trials with dosages given in vg/kg body weight, the dose was calculated for an average body weight of adult person (70 kg).

Targeted delivery has a maximum dosage of 7.5 × 10^15^ viral genome (vg), whereas systemic delivery can go up to 1.5 × 10^17^ vg (Figures 6A,C). For minimum dosages, targeted delivery can go as low as 5.8 × 10^9^ vg, while systemic delivery uses at least 3.5 × 10^13^ vg. These numbers are perhaps unsurprising as higher dosages are needed to compensate for the dilution of systemic administration. However, the large range of dosages used in both delivery routes indicates that effective dosages remain elusive. Overall, 62% of systemically delivered AAVs are dosed at 10^14^-10^16^ vg, whereas 54% of targeted delivered are dosed at 10^11^-10^13^ vg (Figures 6B,D).

### Immunomodulation and AAV

#### Neutralising Antibodies (NAbs)

**Figure 7:**
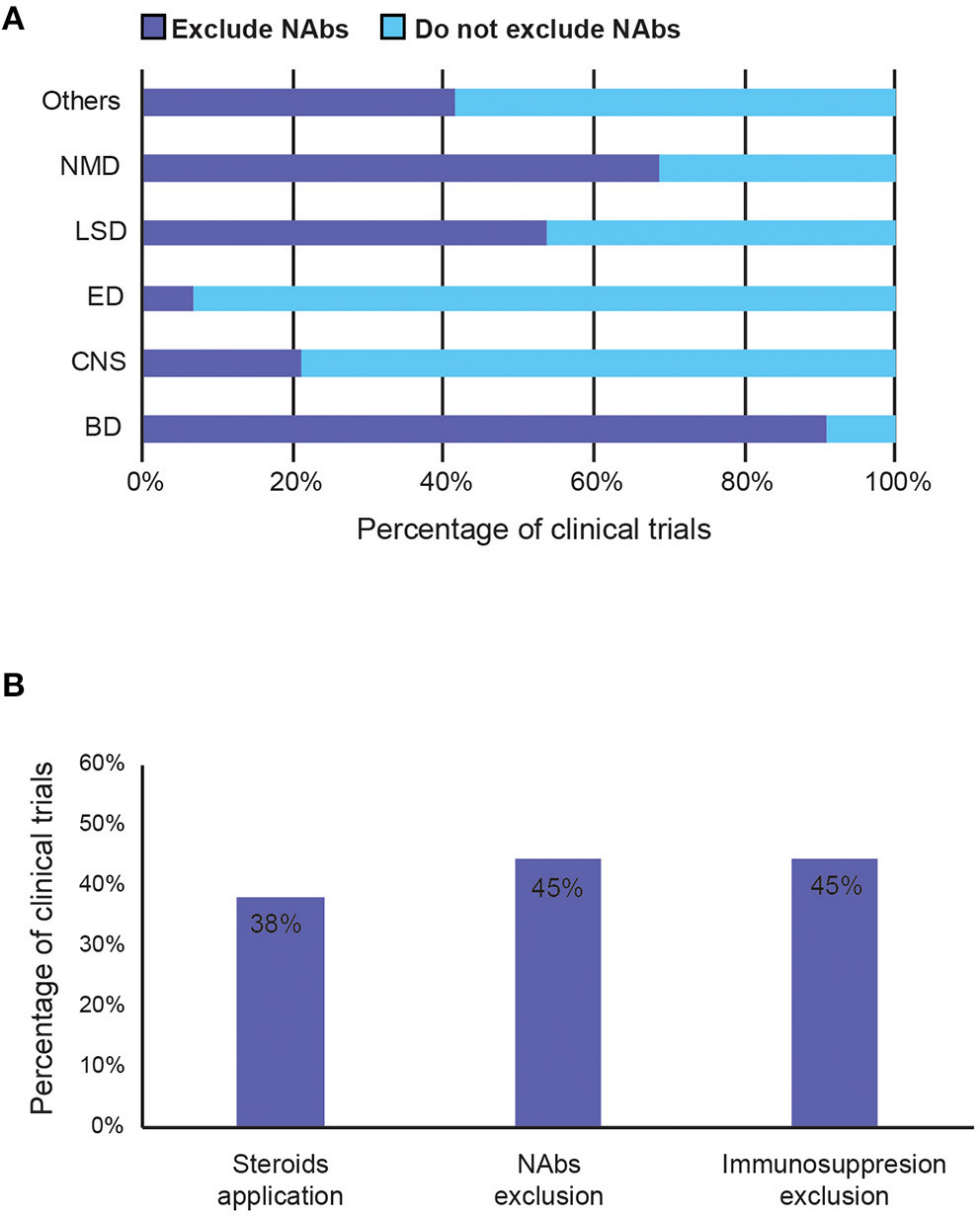
Summary of strategies to avoid immunogenicity induced by AAV vector in clinical trials. **(A)** Percentage of trials that exclude patients with pre-existing anti-capsid NAbs by therapeutic area. **(B)** Percentage of trials that reported (1) potential use of steroids during the study period, (2) exclude patients with pre-existing anti-AAV capsid neutralising antibodies (NAbs), and (3) exclude immunosuppressed patients (such as those with concurrent use of immunosuppressants or with immunosuppressive disorders). BD, Blood disorders; CNS, Central Nervous System; ED, Eye Disorders; LSD, Lysosomal storage disorders; NMD, Neuromuscular Disorders.

To avoid immune-mediated toxicities, patients with pre-existing NAbs against the AAV capsid are often excluded from clinical studies (57). Forty-five percentage of trials exclude patients with pre-existing anti-capsid antibodies (NAbs), presumably to avoid them blocking efficient AAV transduction of the same serotype. However, the percentage varies considerably between therapeutic areas. Nearly 90% of the trials for blood disorder exclude patients with NAbs, whereas only <10% of the trials for eye and 21% of CNS disorders do so, indicating NAbs are of concern rather for systemic delivery than for targeted, particularly in tissues protected by the blood-brain barrier (Figure 7A).

#### Immunosuppression

Immunomodulation regimes aim to prevent the cellular immune response to AAVs, mediated by T cells. They are generally poorly recorded in the trials, even though 38% of studies suggested the potential use of immunosuppressants (Figure 7B). Most immunosuppressants used are mostly just described as systemic or oral corticosteroid, steroids, or glucocorticoids. Specific examples include prednisolone, prednisone, methylprednisolone (Solupred®), sirolimus, tacrolimus (Prograf®, Modigraf®), mycophenolate mofetil (Cellcept®), and cyclosporine. In addition to the listed immunosuppressants co-delivered with the AAV, Figure 7B also includes studies that exclude patients with contraindication to certain immunosuppressants, or those currently taking them, and those that allow use of immunosuppressants if needed. Forty-five percentage of trials also exclude those currently on (chronic) immunosuppressive therapy, immunotherapy, or having immunosuppressive disorders. Although the exact usage of immunosuppression and its effects on clinical outcome are mostly unknown, due to poor recording and unpublished results, 38% is a relatively high number that implies that it may have a perceived value in preventing anti-AAV immunogenicity.

## Discussion

**Figure 8:**
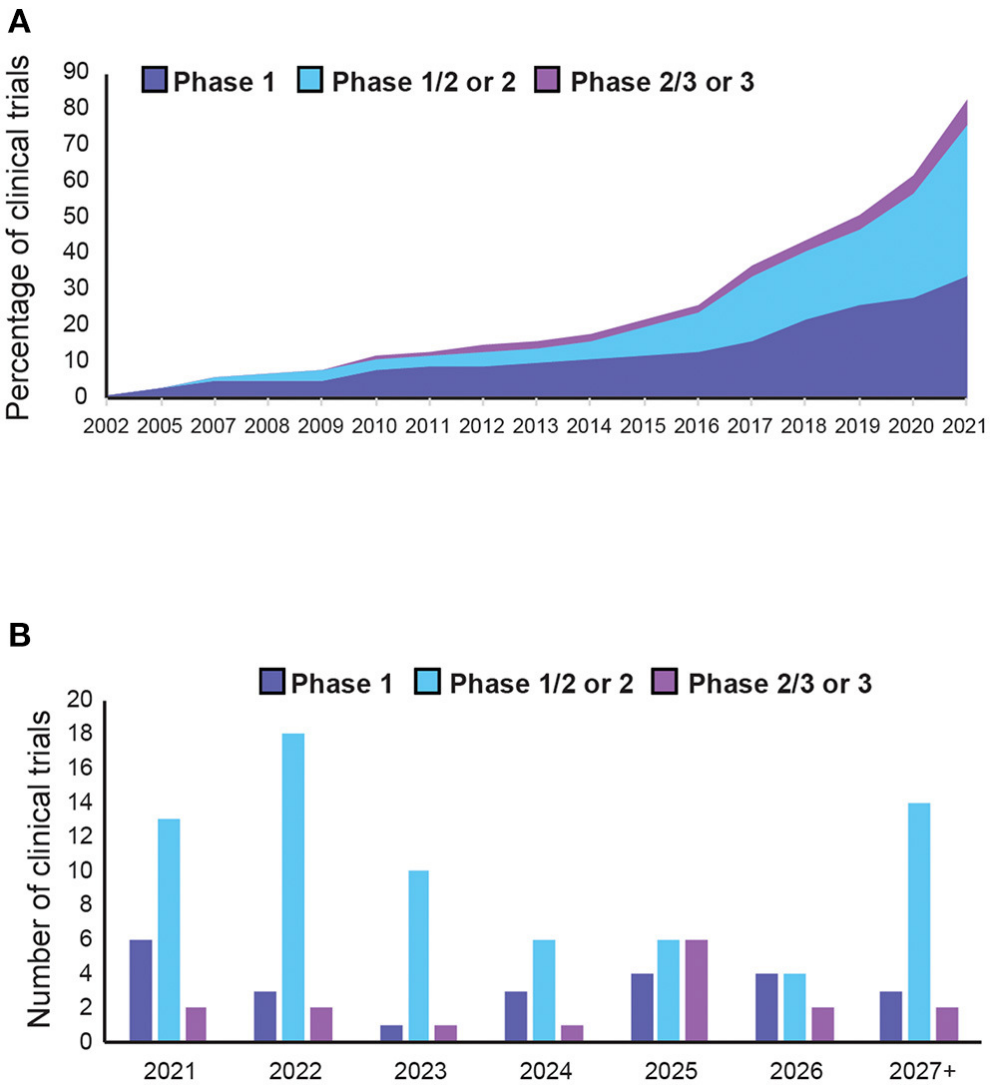
Trends in adeno-associated vector (AAV)-mediated gene therapy in clinical trials. **(A)** Cumulative AAV-mediated gene therapy clinical trials completed in the past 20 years. **(B)** Expected number of of clinical trial completions in the next 5 years or more (see Supplementary Table 5 for details).

Although a wealth of trials are underway, AAV-mediated gene therapy has not yet reached maturity in clinical applications, with only two commercialised products and 75% of trials still at early stage. Nonetheless, the market is set to expand in the coming years, with increasing development of novel approaches. This growth has been further accelerated by the COVID-19 pandemic, which expanded the infrastructure to produce clinical grade AAVs for vaccination programmes. Moreover, the number of trials per year increased annually from four in 2015 to 21 in 2021 (Figure 8). Since most targeted diseases are rare diseases, with significant unmet needs and high costs of existing treatments, it is not uncommon for drug candidates to be granted fast-track or orphan drug designation, providing commercial incentives for developments in this area. Out of all the indications, Haemophilia A is the most targeted disease since it has the maximum number of trials, mostly in late phase development, with higher chances of reaching commercialisation. Most trials for AAV-based gene therapy will be launched after 2021. We can expect over 50 trials reaching completion in the next 3 years, with over 80% of them being phase 2 or above (Figure 8B). This will provide more efficacy data to inform about the prospects for successful new gene therapy candidates in human applications. However, there are still key areas that need to be better understood, possibly by conducting research in non-human primates, which are discussed as below.

### AAV Vector Elements

#### Capsid Engineering

As summarised in Figure 4A, Supplementary Table 3, novel capsids have not yet been widely adopted in clinical settings, despite many being explored in pre-clinical studies. Overall, the best characterised AAV2 is still being used the most, despite having the highest prevalence of NAbs (72% as of 2015) (26). Other studies have also shown that the broad tropism of natural serotypes might not be the most efficient way to deliver vectors, with preclinical efficacies not being replicated in humans. For example, AAV8 has been used in 40% of trials in blood disorders, which conferred stable FIX expression in one study. However, it transduces human hepatocytes 20-times less efficiently than mouse hepatocytes (37). The novel vector, LK03, has been generated to address this issue (37). However, a later study done on over 300 UK patients has revealed that the overall immunoglobulin (Ig)G seroprevalence for AAV-LK03 was 39% in human samples, along with the prevalence of NAbs of 23%, compared to 18% for AAV8 (57). In CNS disorders, although most serotypes studied transduce neurons efficiently, transduction of astrocytes, oligodendrocytes, or microglia is limited (8). AAV/Olig001, the first AAV vector that exhibits strong striatal and oligodendroglial tropism, is a good start in this direction. Nonetheless, this highlights the need for novel capsid variants which improve transduction efficiency while reducing immunogenicity in humans across all therapeutic areas.

#### Tissue-Specific and Endogenous Promoters

Although off-target transduction is one of the leading causes for low transgene expression and toxicity, classic ubiquitous promoters are still used the most. A recent review concluded that 45% of clinical trials used one of CAG, CBA and CMV, as of 2019 (58). That percentage rose to 50% in our analysis (as of April 2021), showing that the field is still very conservative and prefers well-established options. This raises concern as studies have shown that the CMV enhancer, used in both CAG and CMV promoters, can be methylated in CpG dinucleotides over time, both *in vitro* and *in vivo*, silencing the viral transgene it controls (59, 60). Another problem with using strong synthetic promoters is that these could overexpress the transgene and compete with normal expression of other genes, thereby compromising cell health, or resulting in cell stress and transgene clearance. Moving forward, we should look to use more tissue-specific and endogenous promoters to achieve high and sustained transgene levels in humans.

Moreover, different therapeutic areas are exploring tissue-specific promoters at different speeds, with CNS disorders lagging behind especially. This slow development in CNS disorders might be attributed to the BBB in preventing systemic leakage following targeted delivery, compensating for the need for tissue specificity. This is in stark contrast to gene therapy in blood disorders, in which all but one of the disclosed promoters are tissue-specific, likely due to the systemic administration route increasing the need for targeted tissue expression. Indeed, a positive correlation between tissue-specific promoters and systemic delivery (*r* = 0.75), as well as between ubiquitous promoters and targeted delivery (*r* = 0.81) were found in this study. This highlights the opportunity for further improvement in transduction efficiency in CNS disorders by combining tissue-specific vectors and promoters with targeted delivery.

#### Transgene Optimisation

Modifying the transgene to produce more-effective therapeutic proteins is another method to compensate for low transduction efficiency. The FVIII-V3 variant used to treat Haemophilia A in clinical trials has shown a 2-fold increase in transduction potency in mice (61). Additionally, FIX-Padua, a variant of FIX with a hyperactivating R338L mutation, resulted in more efficient thrombin generation by up to 5–10-fold in haemophilia B patients (62).

Another limitation of the AAV cassette is its low capacity of 4.7 kb of DNA. Any larger transgene would not be able to fit into the cassette, limiting the choice of the insert (9). To circumvent this, gene truncation and dual vector strategies have been used in clinical trials. Gene truncation involves removing portions not required for biological activity and appears to be a promising strategy. Key examples include mini-dystrophin for muscular dystrophy and a B-domain deleted form of FVIII (BDD-FVIII) for haemophilia A (24, 63). In dual-vector strategies, two gene segments are co-delivered in different cassettes to allow self-assembly *in vivo* (56, 64). Large genes can also be rescued at the mRNA level by synthetic antisense oligonucleotides or splice site inhibitors, such as anti-HCV shRNA and an exon 2 inhibitor of Acetyl-CoA carboxylase (accA) (65). The increasing popularity of the above approaches in clinical trials provides a promising platform to overcome the limits in AAV packaging capacity. Advances in structural biology allow pharmacologically-important domains of a protein to be identified, opening avenues for other larger genes from other diseases to be considered in AAV gene therapy in the future.

### Standardising Dosing Regime

The large range of dosages used currently in clinical trials indicates that effective dosages remain elusive. In addition, the reported dosing regimes do not include any information related to either viral preparations (for example the ratio of full-to-empty capsids) nor to viral titering characteristics (e.g., assays used to determine neutralising antibody titers). The most-used dosages reported here merely suggest “safe options,” and are not necessarily the optimal dosages. The lack of dose-dependent safety and efficacy data means that there is little evidence as to whether patients are being underdosed or overdosed, which can limit transduction efficiency and result in avoidable toxicity, respectively. However, the optimal dosage is specific for each AAV application, as it depends on many factors ranging from the route and location of drug delivery, AAV variant, to the transgene itself. Therefore, more controlled dose-dependent studies, focussing on the transduction efficiency and immunogenicity, should be carried out in non-human primates to obtain the optimal dosages for different AAV variants. Such data could be used to produce guidelines to inform future trials on the best dosing regimens for selected AAVs, maximising therapeutic effects and ultimately the clinical success of AAV gene therapy.

This is a very important issue in the light of recent developments during the ASPIRO clinical trial, that used an AAV8 based gene therapy to deliver a therapeutic MTM1 (myotubularin) gene in children with X-linked myotubular myopathy (XLMTM). Unfortunately, two out of 17 boys who received AT132 intravenously, at the dose of 3 × 10^14^ vg/kg, developed fatal liver dysfunction. It should be noted that those two boys received a much higher dose (4.80 × 10^15^-7.74 × 10^15^ total vg) due to their weight. It is not yet clear whether their pre-existing conditions or a higher AAV dose triggered liver dysfunction (66).

A better cell targeting, using improved delivery methods, can also reduce the dosage regime. For example, in Eye Disorders, suprachoroidal space (SCS) injection is a relatively new administration method to deliver drugs directly into the posterior segment of the eye, which was shown to enhance treatment effects and minimise side effects in many pre-clinical studies. REGENXBIO has recently initiated the first clinical trial for SCS delivery of RGX-314 in September 2020. Alternatively, in CNS disorders, magnetic resonance imaging helps target brain regions more precisely during surgery, while convection-enhancement helps deliver the drug past the BBB, in a targeted and safe manner, by generating a pressure gradient at the tip of an infusion catheter (67). These new methods are currently being used in five clinical trials, and can be expected to become more widely used in upcoming trials.

### Immunomodulation Strategies With Uncertain Clinical Effectiveness

Currently, the most common way to avoid immunogenicity caused by AAV is to exclude patients with pre-existing NAbs against the AAV capsid used and co-deliver immunosuppressants to prevent T-cell responses. Other strategies suggested in the literature such as elimination of antibody-producing cells by pharmacological means, depleting NAbs prior to AAV administration via plasmapheresis, and chemical shielding of AAV antigens, were not yet seen in clinical applications and should be further explored (2, 68). Alternatively, there is a novel approach to clear NAbs ahead of AAV delivery by using an IgG-degrading enzyme, IdeZ. A recently-published pre-clinical study showed that recombinant IdeZ enzyme efficiently cleared IgG in various species and rescued AAV transduction (69).

#### NAbs Exclusion

Given that pre-existing NAbs were listed as one of the biggest challenges, it was rather surprising that only less than half of the trials exclude patients carrying them. Interestingly, while over 90% of trials for blood disorders exclude NAbs, immune-privileged areas such as the eye and the CNS have merely 7 and 21% exclusion, respectively. Although AAV-mediated gene therapy seems to be relatively well-established in haemophilia, the high exclusion rate means that many patients are ineligible to receive the treatments. Hence, it is vital to better understand effects of NAbs and if necessary to develop new strategies to combat NAbs. The first cardiac gene therapy trial to enrol NAb-positive patients was carried out in 2020, to investigate the influence of pre-existing NAbs on AAV1 (NCT00534703). However, only one out of five patients recruited was NAb-positive, in contrast to much higher rates (59%) of detectable NAbs in the general population. While there were no safety concerns in the NAb-positive patient, T cell responses due to previous exposure were expected. However, the low AAV dosage administered meant that gene transduction was low or absent in other patients, so it is uncertain whether the effect was due to the AAV intervention (70). This trial was terminated early by trial committee recommendation, but further studies with larger sample sizes and higher doses should be conducted to generate more definitive data for the effects of NAbs on the safety and efficacy of AAV-mediated gene transfer. Perhaps to answer this question, BioMarin Pharmaceutical is currently conducting a trial for Valoctocogene Roxaparvovec in 10 Haemophilia A patients with pre-existing NAbs against AAV5 (NCT03520712), in parallel to a trial without NAbs (NCT04684940). Although it will not be completed until 2026, such safety and efficacy results will inform whether stringent exclusion criteria are actually needed.

By contrast, it appears that the blood-brain and blood-eye barrier allow the CNS and eye to be spared from systemic immunity, hence there is no need for exclusion of patients with NAbs. This might also explain the apparent reluctance to explore novel capsids and tissue-specific promoters in such trials. However, depending on the delivery methods, systemic leakage can occur in the CNS too. Poor target delivery and leakage into the cerebrospinal fluid have resulted in adverse events and poor therapeutic efficacies in phase 2 clinical intracerebroventricular GDNF protein delivery (71). Therefore, although CNS and eye appear to be the most promising area to target, such results re-emphasise the need for novel vectors, tissue-specific promoters, and improved delivery methods. It has to be emphasised that some AAV-specific antibodies have been detected at birth, suggesting vertical transmission of maternal antibodies (72). Patients excluded can be rescreened for eligibility at regular intervals during the first year of life, when maternal antibody titres start to decrease, and most humans are seronegative for most serotypes. However, this time window is quite narrow and only applies to diseases affecting infants, such as lysosomal storage disorders (30).

#### Immunosuppressants

The use of immunosuppressants in clinical trials is extremely poorly recorded, with a lack or inconsistency in published results meaning that effective strategies to prevent anti-capsid cellular immunogenicity remain elusive. Although immunosuppression by using oral steroids has successfully limited the loss of transgene expression in several liver-directed AAV gene therapies, results in other trials suggested steroids are insufficient to prevent transgene loss (73). This remains a gap in knowledge that requires more clinical data to draw conclusions and direct design of future clinical trials.

#### Capsid Engineering

There are key examples of AAV2.7m8 and Anc80 capsids, which were selected for their resistance to neutralising with antibodies against AAV2 and AAV8, respectively. In pre-clinical studies, escape mutants for NAbs have been developed by directed evolution, site-directed mutagenesis and rational design, in which critical antigenic sites on the AAV capsid were mutated to evade NAbs in human serum. Examples include AAV2.15, AAV2.4 and AAVhum.8, which all showed more efficient gene transduction than wild-type capsid, while retaining tissue tropism in the presence of anti-AAV serum *in vitro* (74, 75).

#### Improved Manufacturing Capacity to Increase Vector Concentration

Although not a major challenge yet, the ongoing Covid-19 pandemic has drawn huge attention to nucleic acid vaccines, along with enormous funding to increase manufacturing capacities of viral vectors. Recently, two AAV-based vaccine candidates have shown pre-clinical potency, holding promise for a single-dose vaccine that is stable at room temperature. Mass General Brigham's candidate, AAVCOVID, uses a hybrid serotype capsid AAVrh32.33, to which no relevant pre-existing immunity exists in humans, to deliver the antigenic spike protein of SARS-CoV2 to induce immune response against the virus (76). Novartis recently announced that it is going to manufacture AAVCOVID for clinical trials, using its existing facility, indicating that there is a growing demand for AAV production, indirectly benefiting its usage in gene therapy (77).

## Conclusion

While AAVs are well-studied in pre-clinical settings, translatability into clinical use in humans has been relatively limited. This research summarised current key approaches and identified a number of challenges in using AAVs for gene therapy. Based on the trends and knowledge gaps revealed, future research should focus on developing optimal dosing regimes based on dose-dependent toxicity and efficacy studies, especially in non-human primates. It is becoming apparent that engineering new capsid variants by rational design and directed evolution will improve transduction efficiency and reduce immunogenicity in all therapeutic areas, which is especially important for systemically delivered drugs. This should be of importance for CNS drug development where the majority of the trials are using AAV2 capsids that potentially limit clinical effectiveness. Similarly, the choice of the promoter could be critical for the success of gene therapy and avoiding those promoters that could be silenced by methylation (e.g., CMV and CAG) should be a priority. There is also an urgent need to establish guidelines for inclusions/exclusions of NAbs patients and for the use of immunosuppressants in clinical trials.

## Data Availability Statement

The original contributions presented in the study are included in the article/Supplementary Material, further inquiries can be directed to the corresponding author/s.

## Author Contributions

MI and MM conceived and designed the experiments. HKEA performed the experiments and analysed the data. HKEA, MI, and MM wrote the manuscript. All authors contributed to the article and approved the submitted version.

## Funding

This work was supported by Imperial/ICR/NIHR BRC/NHS Confidence in Concept (iCiC) grant. MI is funded by Investigator award (No. WT102944) from the Wellcome Trust U.K. These funders had no role in study design, data collection and analysis, decision to publish or preparation of the manuscript.

## Conflict of Interest

The authors declare that the research was conducted in the absence of any commercial or financial relationships that could be construed as a potential conflict of interest.

## Publisher's Note

All claims expressed in this article are solely those of the authors and do not necessarily represent those of their affiliated organizations, or those of the publisher, the editors and the reviewers. Any product that may be evaluated in this article, or claim that may be made by its manufacturer, is not guaranteed or endorsed by the publisher.
