# Supplementary material for: Gene Therapy Advances: A Meta-Analysis of AAV Usage in Clinical Settings
Source: Front Med (Lausanne). 2022 Feb 9;8:809118. doi: 10.3389/fmed.2021.809118 (PMC8864161; doi:10.3389/fmed.2021.809118)
Supplement: Supplementary file 1 [file Data_Sheet_1.PDF]

Supplementary Materials

Gene therapy advances: a meta-analysis of AAV usage in clinical settings

Hau Kiu Edna Au, Mark Isalan, Michal Mielcarek

Supplementary Figure 1. Distribution of AAV capsid in the specific therapeutic areas. BD (Blood disorders); CNS (Central Nervous System); ED (Eye Disorders); Lysosomal storage disorders (LSD); Neuromuscular Disorders (NMD).

Supplementary Figure 2. Distribution of promoters used in clinical trials per specific therapeutic area. BD (Blood disorders); CNS (Central Nervous System); ED (Eye Disorders); Lysosomal storage disorders (LSD); Neuromuscular Disorders (NMD).

This file includes:

Supplementary Table 1. Adeno-associated virus (AAV) gene therapy trials in the analysis dataset.
Supplementary Table 2. Capsid description.
Supplementary Table 3. Promoter abbreviations.
Supplementary Table 4. Transgene abbreviation.

Supplementary Table 5. Background and progression of AAV clinical trials.

REFERENCES

Supplementary Table 1 | Adeno-associated virus (AAV) gene therapy trials in the analysis dataset (duplicates included)

Therapeutic area	Indication	Drug ID	Vector	Promoter	Transgene	Mode of action	Delivery route	Target tissue	Exclude NAbs	Exclude immunosuppressed patients	Phases	NCT Number
**Blood Disorders (BD)**	Hemophilia A	rAAV2/6-hFVIII	AAV2/6	liver-specific	FVIII	Replacement	Systemic	Hepatocytes	Y	N	Phase 3	NCT04370054
		SB-525	AAV2/6	liver-specific	FVIII-SQ	Replacement	Systemic	Hepatocytes	Y	N	Phase 2	NCT03061201
		AAV2/8-HLP-FVIII-V3	AAV2/8	HLP	FVIII-V3	Replacement	Systemic	Hepatocytes	Y	N	Phase 1	NCT03001830
		DTX201	AAVhu37	liver-specific	FVIII-SQ	Replacement	Systemic	Hepatocytes	Y	N	Phase 1 / 2	NCT03588299
		BAX 888	AAV2/8	TTR	FVIII-SQ	Replacement	Systemic	Hepatocytes	Y	N	Phase 1 / 2	NCT03370172
		Valoctocogene roxaparvovec	AAV5	HLP	FVIII-SQ	Replacement	Systemic	Hepatocytes	Y	Y	Phase 1 / 2	NCT02576795
		SPK-8016	LK03	AAT	FVIII	Replacement	Systemic	Hepatocytes	Y	N	Phase 1 / 2	NCT03734588
		SPK-8011	LK03	CBA	FVIII-SQ	Replacement	Systemic	Hepatocytes	Y	N	Phase 1 / 2	NCT03003533
		ASC618	N/A	liver-specific	FVIII-SQ	Replacement	Systemic	Hepatocytes	Y	Y	Phase 1 / 2	NCT04676048
		GS001	N/A	N/A	FVIII	Replacement	Systemic	Hepatocytes	Y	N	N/A	NCT04728841
	Hemophilia B	AAV-FIX	AAV2	N/A	hFIX	Replacement	Systemic	Hepatocytes	Y	N	Phase 1 / 2	NCT00076557
		scAAV2/8-LP1-hFIXco	AAV2/8	LP1	hFIX	Replacement	Systemic	Hepatocytes	Y	N	Phase 1	NCT00979238
		AMT-061	AAV5	liver-specific	hFIX-Padua	Replacement	Systemic	Hepatocytes	N	N	Phase 3	NCT03569891
		AAV8-hFIX19	AAV8	AAT	hFIX19	Replacement	Systemic	Hepatocytes	Y	N	Phase 1	NCT01620801
		SHP648	AAV8	TTR	hFIX	Replacement	Systemic	Hepatocytes	Y	N	Phase 1 / 2	NCT04394286
		AskBio009	AAV8	TTR	hFIX-Padua	Replacement	Systemic	Hepatocytes	Y	N	Phase 1 / 2	NCT01687608
		DTX101	AAVrh10	liver-specific	hFIX	Replacement	Systemic	Hepatocytes	Y	N	Phase 1 / 2	NCT02618915
		FLT180a	AAVS3	liver-specific	hFIX-Padua	Replacement	Systemic	Hepatocytes	N	N	Phase 1	NCT03369444
		BBM-H901	N/A	N/A	hFIX	Replacement	Systemic	Hepatocytes	Y	N	N/A	NCT04135300
		SPK-9001	Spark100	liver-specific	hFIX-Padua	Replacement	Systemic	Hepatocytes	Y	N	Phase 3	NCT03587116
		SB-FIX	N/A	liver-specific	hFIX	Editing	Systemic	Hepatocytes	Y	Y	Phase 1	NCT02695160
	HoFH	AVV-hLDLR	NAV AAV8	TBG	hLDLR	Replacement	Systemic	Hepatocytes	Y	N	Phase 1 / 2	NCT02651675
**Central Nervous**	Alzheimer	AAV-hTERT	N/A	N/A	hTERT	Addition	Systemic	Brain	N	N	Phase 1	NCT04133454

**System Disorders (CNS)**	Disease	LX1001	AAVrh.10	CAG	APOE2	Addition	Targetted	CNS	Y	Y	Phase 1	NCT03634007
		CERE-110	AAV2	CAG	NGF	Addition	Targetted	Nucleus basalis of Meynert	N	Y	Phase 2	NCT00876863
	Frontotemporal Dementia	PR006	AAV9	CAG	GBA1/PGRN	Replacement	Targetted	Brain	N	N	Phase 1 / 2	NCT04408625
		PBFT02	AAV2/1	CAG	GRN	Replacement	Targetted	Brain	N	N	Phase 1 / 2	NCT04747431
	Huntington Disease	rAAV5-miHTT	rAAV5	CAG	miHTT	Silencing	Targetted	Brain	N	N	Phase 1 / 2	NCT04120493
	Multiple System Atrophy	AAV2-GDNF	AAV2	CMV	GDNF	Addition	Targetted	Putamen	N	N	Phase 1	NCT04680065
	Parkinson's Disease	AAV2-GDNF	AAV2	CMV	GDNF	Addition	Targetted	Putamen	N	N	Phase 1	NCT04167540
		AAV2-GDNF	AAV2		GDNF	Addition	Targetted	Brain	Y	Y	Phase 1	NCT01621581
			rAAV2	CMV	hAADC-2	Replacement	Targetted	Brain	N	Y	Phase 1 / 2	NCT02418598
		AAV-hAADC-2	rAAV2	CMV	hAADC	Replacement	Targetted	Brain	Y	N	Phase 1	NCT00229736
		VY-AADC01	AAV2	CMV	hAADC	Replacement	Targetted	Putamen	Y	Y	Phase 1	NCT01973543
		VY-AADC01	AAV2	CMV	hAADC	Replacement	Targetted	Putamen	N	Y	Phase 1	NCT03065192
		VY-AADC02	AAV2	CMV	hAADC	Replacement	Targetted	Putamen	N	N	Phase 2	NCT03562494
		PR001A	AAV9	SYN1	GBA1	Replacement	Targetted	Brain	N	Y	Phase 1 / 2	NCT04127578
		AAV-GAD	AAV2	NSE	GAD	Addition	Targetted	Subthalamic nucleus	N	N	Phase 2	NCT00643890
		CERE-120	AAV2	CAG	NTN	Addition	Targetted	Putamen	N	Y	Phase 2	NCT00400634
	Sanfilippo Syndrome B	rAAV2/5-hNAGLU	rAAV2/5	mPKG	hNAGLU	Replacement	Targetted	Brain	N	N	Phase 1 / 2	NCT03300453
	Canavan Disease	rAAV-Olig001-ASPA	AAV/Olig001	CBA	ASPA	Replacement	Targetted	Oligodendrocytes	N	N	Phase 1 / 2	NCT04833907
**Eye Disorders (ED)**	Achromatopsia	AAV2/8-hCARp.hCNGB3	AAV2/8	hCAR	CNGB3	Replacement	Targetted	Retina	N	N	Phase 1 / 2	NCT03001310
		AAV- CNGA3	AAV2/8	hCAR	CNGA3	Replacement	Targetted	Retina	N	N	Phase 1 / 2	NCT03758404
		rAAV2tYF-PR1.7-hCNGA3	rAAV2tYF	PR1.7	hCNGA3	Replacement	Targetted	Retina	N	N	Phase 1 / 2	NCT02599922
		rAAV2tYF-PR1.7-hCNGB3	rAAV2tYF	PR1.7	hCNGB3	Replacement	Targetted	Retina	N	N	Phase 1 / 2	NCT02935517
	Choroideremia	AAV-REP1	AAV2	CAG	REP1	Replacement	Targetted	Retina	N	N	Phase 2	NCT02407678
		AAV2-REP1	AAV2	N/A	REP1	Replacement	Targetted	Retina	N	N	Phase 2	NCT02553135
		BIIB111	AAV2	N/A	REP1	Replacement	Targetted	Retina	N	N	Phase 3	NCT03496012

		rAAV2.REP1	AAV2	CAG	REP1	Replacement	Targetted	Retina	N	N	Phase 1 / 2	NCT01461213
		4D-110	4D-R100	CAG	CHM	Replacement	Targetted	Retina	Y	N	Phase 1	NCT04483440
		AAV2-hCHM	AAV2	CBA	CHM	Replacement	Targetted	Retina	N	N	Phase 1 / 2	NCT02341807
	Leber Congenital Amaurosis	Luxturna	AAV2	CAG	hRPE65v2	Replacement	Targetted	Retina	N	N	Phase 3	NCT00999609
		AAV RPE65	AAV2/5	NA65p	RPE65	Replacement	Targetted	Retina	N	N	Phase 1 / 2	NCT02781480
		tgAAG76	rAAV 2/2	hRPE65p	hRPE65	Replacement	Targetted	Retina	N	N	Phase 1 / 2	NCT00643747
		rAAV2-CB-hRPE65	AVV2/2	CBA	hRPE65	Replacement	Targetted	Retina	N	Y	Phase 1 / 2	NCT00749957
		GS010	rAAV2/2	CMV	ND4	Replacement	Targetted	Retina	N	N	Phase 3	NCT03293524
	Age-related Macular Degeneration	GT005	AAV2	CAG	CFI	Addition	Targetted	Retina	N	N	Phase 1 / 2	NCT03846193
		AAV2-sFLT01	AAV2	CBA	sFLT01	Addition	Targetted	Retina	N	Y	Phase 1	NCT01024998
		rAAV.sFlt-1	AAV2	CMV	sFlt-1	Addition	Targetted	Retina	N	N	Phase 1 / 2	NCT01494805
		RGX-314	NAV AAV2/8	CB7	anti-VEGF Fab	Addition	Targetted	Retina	N	N	Phase 2 / 3	NCT04704921
		ADVM-022	AAV2.7m 8	CMV	aflibercept	Addition	Targetted	Retina	N	N	Phase 2	NCT04418427
	Retinitis Pigmentosa	AAV2/5-hPDE6B	AAV2/5	hRK	hPDE6B	Replacement	Targetted	Retina	N	Y	Phase 1 / 2	NCT03328130
		rAAV.hPDE6A	AAV2/8	hRHO	hPDE6A	Replacement	Targetted	Retina	N	Y	Phase 1 / 2	NCT04611503
		GS030-DP	rAAV2.7m 8	CAG	ChrimsonR-tdTomat	Addition	Targetted	Retina	N	N	Phase 1 / 2	NCT03326336
		AAV2/5-RPGR	AAV2/5	hGRK1	RPGR	Replacement	Targetted	Retina	N	N	Phase 3	NCT04671433
		BIIB112	AAV2/8	hGRK1	RPGR	Replacement	Targetted	Retina	N	N	Phase 1 / 2	NCT03116113
		4D-125 IVT	4D-R100	CAG	RPGR	Replacement	Targetted	Retina	N	N	Phase 1 / 2	NCT04517149
		rAAV2tYF-GRK1-RPGR	AAV2tYF	GRK1	hRPGR	Replacement	Targetted	Retina	N	Y	Phase 2 / 3	NCT04850118
		rAAV	rAAV2	VMD2	hMERTK	Addition	Targetted	Retina	Y	Y	Phase 1	NCT01482195
	Retinoschisis	AAV8-scRS/IRBPhRS	AAV8	hRS1	RS1	Replacement	Targetted	Retina	N	Y	Phase 1 / 2	NCT02317887
		rAAV2tYF-CB-hRS1	AAV2tYF	CAG	hRS1	Replacement	Targetted	Retina	N	N	Phase 1 / 2	NCT02416622
**Lysosomal Storage Disorders (LSD)**	Batten Disease	AAVrh.10CUhCLN2	AAVrh.10	CAG	hCLN2	Replacement	Targetted	Brain	N	Y	Phase 1 / 2	NCT01414985
		AAV2CUhCLN2	AAV2	CAG	hCLN2	Replacement	Targetted	Brain	N	N	Phase 1	NCT00151216
		AT-GTX-501	scAAV9	CBA	CLN6	Replacement	Targetted	Lumbar spinal cord	Y	N	Phase 1 / 2	NCT02725580

		AT-GTX-502	scAAV9	CMV	CLN3	Replacement	Targetted	Lumbar spinal cord	Y	Y	Phase 1 / 2	NCT03770572
		AAV9/CLN7	AAV9	N/A	CLN7/MFSD8	Replacement	Targetted	Lumbar spinal cord	N	Y	Phase 1	NCT04737460
	Familial Lipoprotein Lipase Deficiency	AMT-011	AAV1	CMV	LPL[S447X]	Replacement	Targetted	Vascular endothelial	N	Y	Phase 2 / 3	NCT00891306
	GM1 Gangliosidosis	LYS-GM101	AAVrh.10	CAG	GLB1	Replacement	Targetted	CNS	N	Y	Phase 1 / 2	NCT04273269
		AAV9-GLB1	AAV9	CBA	GLB1	Replacement	Systemic	Nerve cells	Y	Y	Phase 1 / 2	NCT03952637
	GM2 Gangliosidosis	TSHA-101	AAV9	CAG	HEXBP2A-HEXA	Replacement	Targetted	Brain	N	Y	Phase 1 / 2	NCT04798235
	Tay-Sachs/Sandhoff Disease	AXO-AAV-GM2	AAVrh8	CMV	HEXA or HEXB	Replacement	Targetted	Cerebrospinal fluid	N	Y	Phase 1	NCT04669535
	Gaucher Disease	PR001	AAV9	SYN1	GBA1	Replacement	Targetted	Brain	N	Y	Phase 1 / 2	NCT04411654
	Fabry Disease	ST-920	AAV2/6	liver-specific	hGLA	Replacement	Systemic	Multi-organ	Y	Y	Phase 1 / 2	NCT04046224
		4D-310	4D-C102	CAG	GLA	Replacement	Systemic	Multi-organ	Y	Y	Phase 1 / 2	NCT04519749
		FLT190	AAVS3	FRE1	αGLA	Replacement	Systemic	Multi-organ	N	N	Phase 1 / 2	NCT04040049
	MPS I	SB-318	AAV2/6	Albumin	IDUA	Editing	Systemic	Albumin gene locus in the genome of liver cell	Y	N	Phase 1 / 2	NCT02702115
	MPS II	SB-913	AAV 2/6	Albumin	IDS	Editing	Systemic	Hepatocytes	N	Y	Phase 1 / 2	NCT03041324
	MPS IIIA	SAF-301	AAVrh.10	mPGK	SGSH, SUMF1	Replacement	Targetted	Cerebral	N	N	Phase 1 / 2	NCT01474343
		ABO-102	scAAV2/9	mU1a	SGSH	Replacement	Systemic	?	Y	N	Phase 1 / 2	NCT04088734
		LYS-SAF302	AAVrh.10	CAG	SGSH	Replacement	Targetted	Cerebral	N	N	Phase 2 / 3	NCT03612869
	MPS VI	AAV2/8.TBG.hARSB	AAV2/8	TBG	hARSB	Replacement	Systemic	Hepatocytes	Y	Y	Phase 1 / 2	NCT03173521
	Glycogen Storage Disorder 1	DTX401	AAV8	liver-specific	G6Pase-α	Replacement	Systemic	Hepatocytes	Y	N	Phase 1 / 2	NCT03517085
	Pompe Disease	AAV2/8LSPhGAA	AAV2/8	TBG	hGAA	Replacement	Systemic	Hepatocytes	Y	N	Phase 1 / 2	NCT03533673
		rAAV-GAA	rAAV9	DES	hGAA	Replacement	Systemic	Muscles	Y	Y	Phase 1	NCT02240407
		rAAV1-CMV-GAA	rAAV1	CMV	hGAA	Replacement	Targetted	Diaphragm muscle	Y	Y	Phase 1 / 2	NCT00976352
		AT845	AAV8	Liver/DES	hGAA	Replacement	Systemic	Muscles	Y	Y	Phase 1 / 2	NCT04174105
		SPK-3006	(bioengin	liver-specific	hGAA	Replacement	Systemic	Hepatocytes	Y	N	Phase 1 / 2	NCT04093349

			eered)									
**Neuromuscular Disorders (NMD)**	AADC Deficiency	AAV2-hAADC	AAV2	CMV	hAADC	Replacement	Targetted	Substantia nigra	Y	N	Phase 1	NCT02852213
		AAV2-hAADC	AAV2	CMV	hAADC	Replacement	Targetted	Putamen	Y	N	Phase 2	NCT02926066
	Becker Muscular Dystrophy	AAV1.CMV.huFollistatin344	rAAV2/1	CMV	FS344	Addition	Targetted	Thigh muscle	Y	Y	Phase 1	NCT01519349
	Charcot-Marie-Tooth Neuropathy	scAAV1.tMCK.NTF3	scAAV1	tMCK	NTF3	Replacement	Targetted	Leg muscles	Y	Y	Phase 1 / 2	NCT03520751
	Duchenne Muscular Dystrophy	scAAV9.U7.ACCA	scAAV9	U7	U7-ACCA	Silencing	Systemic	Limb	Y	Y	Phase 1 / 2	NCT04240314
		PF-06939926	AAV9	muscle-specific	mini-DMD	Replacement	Systemic	Skeletal and cardiac muscles	Y	Y	Phase 1	NCT03362502
		d3990	rAAV2/5	CMV	mini-DMD	Replacement	Targetted	Biceps muscle	N	Y	Phase 1	NCT00428935
		SGT-001	AAV9	CK8	mini-DMD	Replacement	Systemic	Skeletal and cardiac muscle	N	N	Phase 1 / 2	NCT03368742
		rAAV1.CMV.huFollistin344	rAAV1	CMV	huFollistin344	Addition	Targetted	Lower limbs muscles	Y	N	Phase 1 / 2	NCT02354781
	Dysferlinopathy	rAAVrh74.MHCK7.DYSF.DV	rAAVrh74	MHCK7	DYSF	Replacement	Targetted	Leg muscles	Y	N	Phase 1	NCT02710500
	Krabbe Disease	FBX-101	AAV2/rh10	CMV	GALC	Replacement	Systemic	?	N	N	Phase 1 / 2	NCT04693598
	Limb Girdle Muscular Dystrophy 2C	AAV1-γ-sarcoglycan	AAV1	DES	γ-sarcoglycan	Replacement	Targetted	Carpi radialis muscle	Y	N	Phase 1	NCT01344798
	Muscular Dystrophies	rAAV1.tMCK.hγ-sarcoglycan	rAAV1	tMCK	hαSG	Replacement	Targetted	Muscles	N	Y	Phase 1	NCT00494195
	Spinal Muscular Atrophy	Zolgensma	AAV9	CAG	hSMN	Replacement	Systemic	Motor neurons, peripheral tissues	N	N	Phase 3	NCT03461289
		Zolgensma	AAV9	CAG	hSMN	Replacement	Targetted	Motor neurons	Y	N	Phase 1	NCT03381729
	Myotubular Myopathy	AT132	AAV8	DES	hMTM1	Replacement	Systemic	?	Y	Y	Phase 1 / 2	NCT03199469
**Others: Cardiac**	Critical Limb Ischemia	AAV-hTERT	AAV	N/A	hTERT	Addition	Systemic	?	N	N	Phase 1	NCT04110964
	Danon Disease	RP-A501	AAV9	CAG	LAMP2B	Replacement	Systemic	Heart	Y	N	Phase 1	NCT03882437
	Chronic Heart Failure	AAV1/SERCA2a	AAV1	CMV	SERCA2a	Addition	Targetted	Coronary arteries	N	N	Phase 2	NCT00534703
	Congestive Heart Failure	SRD-001	AAV1	α-MHC	SERCA2a	Addition	Targetted	Coronary arteries	Y	N	Phase 1 / 2	NCT04703842
		MYDICAR	AAV2/1	α-MHC	SERCA2a	Addition	Targetted	Coronary arteries	Y	N	Phase 2	NCT01966887

**Others: Lung**	Alpha-1 Antitrypsin Deficiency	rAAV1-CB-hAAT	rAAV2/1	CAG	hAAT	Replacement	Systemic	Liver	Y	Y	Phase 2	NCT01054339
	Cystic Fibrosis	AAV-CFTR	rAAV2	N/A	CFTR	Replacement	Targetted	Airways	N	N	Phase 1	NCT00004533
**Others: Inflammatory**	Rheumatoid Arthritis	ART-I02	AAV2/5	NF-kB	hIFN-b	Addition	Targetted	Joints	Y	N	Phase 1	NCT02727764
		ART-I02	AAV2/5	NF-kB	hIFN-b	Addition	Targetted	Wrist joint	Y	Y	Phase 1	NCT03445715
		tgAAC94	AAV2	CMV	TNFR:Fc	Addition	Targetted	Joint space	N	Y	Phase 1 / 2	NCT00126724
	Osteoarthritis	sc-rAAV2.5IL-1Ra	sc-rAAV2.5	CMV	IL-1Ra	Addition	Targetted	Knee joint	N	Y	Phase 1	NCT02790723
**Others: Liver**	Acute Intermittent Porphyria	rAAV2/5-PBGD	rAAV2/5	EalbAAT	hPBGD	Replacement	Systemic	Hepatocytes	Y	N	Phase 1	NCT02082860
	Crigler-Najjar Syndrome	AT342	AAV8	N/A	UGT1A1	Replacement	Systemic	Hepatocytes	Y	Y	Phase 1 / 2	NCT03223194
		GNT0003	AAV2/8	ApoE/AAT	hUGT1A1	Replacement	Systemic	Hepatocytes	N	N	N/A	NCT03466463
	Wilson's Disease	VTX-801	Anc80	AAT	ATP7B-minigene	Replacement	Systemic	Hepatocytes	N	N	Phase 1 / 2	NCT04537377
	Hepatitis C	TT-034	AAV2/8	hybrid Pol III	anti-HCV shRNA	Silencing	Systemic	Hepatocytes	Y	Y	Phase 1 / 2	NCT01899092
**Others: Viral**	HIV	gAAC09	rAAV2	N/A	HIV	Addition	Targetted	Serum	N	Y	Phase 2	NCT00888446
		rAAV1-PG9DP	rAAV1	CMV, EF1a	PG9DP	Addition	Systemic	Serum	Y	N	Phase 1	NCT01937455
		VRC-HIVAAV070-00-GT	AAV8	CMV, U6	VRC07	Addition	Systemic	Serum	N	N	Phase 1	NCT03374202
**Others: Metabolic**	Phenylketonuria (PKU)	BMN 307	AAV 5	liver-specific	hPAH	Replacement	Systemic	Hepatocytes	N	N	Phase 1 / 2	NCT04480567
	Ornithine Transcarbamylase Deficiency	scAAV8OTC	AAV8	TBG	OTC	Replacement	Systemic	Hepatocytes	N	N	Phase 1 / 2	NCT02991144
**Others: Cancer**	Radiation Induced Xerostomia	AAV2hAQP1	AAV2	CMV	hAQP1	Addition	Systemic	Parotid gland	N	Y	Phase 1	NCT04043104
		AAV2hAQP1	AAV2	CMV	hAQP1	Addition	Targetted	Parotid gland	N	Y	Phase 1	NCT02446249

Supplementary Table 2 |Capsid Description

Capsid	Characteristics (Tropism; Modification - improved features)	Reference
AAV1	Neuro and muscle tropism	[1]
AAV2	Broad tropism	[1]
AAV5	Neuro and retina tropism	[1]
AAV6	Muscle and lung tropism	[1]
AAV8	Liver, muscle, and heart tropism	[1]
AAV9	Liver, muscle, and lung tropism	[1]
AAVrh.10	Brain tropism;<br>Greater enzyme distribution, better immunogenicity than AAV2	[2]
AAVrh.74	Muscle tropism;<br>Good transduction properties, lower pre-existing human population immunity than AAV2	[3, 4]
AAVhu37	Liver tropism;<br>Favourable biodistribution, durable transgene expression	[5]
AAV2tYF	Retina tropism;<br>Surface tyrosine residues mutated to phenylalanine - stronger and more widespread transgene expression	[6]
AAV2.7m8	Retina tropism;<br>10-amino acid insertion in surface variable region - high transduction efficiency and lower immunogenicity	[7]
AAVS3	Liver tropism;<br>Rationally designed by gene swapping - stronger liver targetting	[8]
AAV/Olig001	Neuro tropism;<br>Capsid shuffled - strongly target striatal oligodendroglial	[9]
Anc80	Retina tropism;<br>Backwards-directed evolution - stronger retinal targetting	[10]
Lk03	Liver tropism;<br>Capsid shuffled - stronger liver targetting	[11]
Spark100	Liver tropism;<br>Sustained transgene expression	[12]
4D-C102	Muscle tropism;<br>Directed evolution - reduced immunogenicity	[13]
4D-R100	Retina tropism;<br>Directed evolution - efficiently transduce all layers of the retina	[14]

Supplementary Table 3 | Promoter abbreviations

Abbreviation	Meaning
**Ubiquitous**	
CAG	Cytomegalovirus (CMV) major immediate-early enhancer element combined with the first intron and first exon of Chicken Beta-Actin promoter (CBA)
CB7	CBA promoter/CMV enhancer
CBA	Chicken Beta-Actin promoter
CMV	Cytomegalovirus promoter
EF-1α	Elongation Factor-1 alpha
NF-kB	Nuclear Factor- Kb
PSE-7	Proximal Sequence Element 7
mPGK	Murine Phosphoglycerate Kinase promoter
U1a	Murine small nuclear RNA promoter
U6	RNA Polymerase III U6 promoter
Liver-Specific	
HLP	Hybrid Human Liver promoter
hAAT	Human A-1 Antitrypsin promoter
LP1	Liver promoter
TBG	Thyroxine-Binding Globulin
TTR	Transthyretin Promoter
**Neuro-specific**	
SYN1	Proximal region of the Synapsin I promoter
NSE	Rat Neuron-Specific Enolase
**Muscle-specific**	
tMCK	Triple tandem copies of Mouse Muscle Creatine Kinase enhancer ligated to its basal promoter
CK8	Mouse Creatine Kinase promoter/enhancer element
MHCK7	Murine Muscle Creatine Kinase (CK) and α-myosin heavy-chain genes
SMN	Survival Motor Neuron promoter
DES	Human Desmin enhancer/promoter
**Retina-specific**	
RK	Human Rhodopsin Kinase
RHO	776 bp-long rho promoter fragment carrying the zf6-cis deletion
hGRK1	Human Rhodopsin Kinase promoter
hCAR	Human Cone Arrestin promoter
hRPE65p	Human retinal pigmented epithelium
P546	Truncated Mecp2-promoter
PR1.7	1.7-Kb L-Opsin Promoter
**Cardiac-specific**	
α-MHC	α-myosin heavy chain promoter

Supplementary Table 4 |Transgene abbreviation

Abbreviation	Meaning
AADC	Aromatic l-amino acid decarboxylase
AAT	Alpha-1 antitrypsin
anti-HCV shRNA	Anti-hepatitis C virus short hairpin RNAs
anti-VEGF Fab	Anti–vascular endothelial growth factor antigen-binding fragment
APOE2	Apolipoprotein E isoform 2
AQP1	Aquaporin-1
ARSB	Arylsulfatase B
ASPA	Aspartoacylase
ATP7B	Copper-transporting ATPase 2
CFI	Human complement factor
CFTR	Cystic fibrosis transmembrane conductance regulator
CHM	CHM Rab Escort Protein
CLN	Ceroid lipofuscinosis
CNGA	One photoreceptor cyclic nucleotide-gated channal
DYSF	Dysferlin
FS344	Follistin344
G6Pase-α	Glucose 6-phosphatase
GAD	Glutamate Decarboxylase 1
GALC	Galactosylceramidase
GBA1	Glucocerebrosidase
GDNF	Glial Cell Derived Neurotrophic Factor
GLA	Galactosidase Alpha
GLB1	β-galactosidase
GRN	Progranulin
HEXA/B	Hexosaminidase A/B
GAA	Acid alpha-glucosidase
HIV	Human immunodeficiency virus
IDS	Iduronate 2-Sulfatase
IDUA	α-L-iduronidase
IFN-b	Interferon beta-1a
IL-1Ra	Interleukin-1 receptor antagonist
LAMP2B	Lysosome-associated membrane protein 2
LPL	Lipoprotein Lipase
MERTK	MER Proto-Oncogene, Tyrosine Kinase
MFSD8	Major Facilitator Superfamily Domain Containing 8
miHTT	MicroRNA targeting huntingtin
DMD	Dystrophin
MTM1	Myotubularin
NAGLU	Alpha-N-acetylglucosaminidase
ND4	NADH dehydrogenase 4
NGF	Nerve Growth Factor
NTF3	Neurotrophin 3
NTN	Neurturin

OTC	Ornithine transcarbamylase
PAH	Phenylalanine hydroxylase
PBGD	Porphobilinogen deaminase
PDE6A/B	Rod cGMP phosphodiesterase 6 subunit A/B
PGRN	progranulin
PG9DP	PG9 Antibody
REP1	Rab escort protein 1
RPE65	Retinal pigmented epithelium
RPGR	X-linked retinitis pigmentosa GTPase regulator
RS1	Retinoschisin 1
SERCA2a	Sarco/endo plasmic reticulum calcium (Ca2+) ATPase cardiac isoform
sFlt-1	Truncated form of the Fms Related Receptor Tyrosine Kinase 1
SGSH	N-sulfoglucosamine sulfohydrolase
SMN	Survival motor neuron
SUMF1	Sulfatase Modifying Factor 1
TERT	Telomerase reverse transcriptase
TNFR:Fc	Fusion of tumor necrosis factor receptor and Fc region of the human immunoglobulin
U7-accA	Non-coding RNA binding exon 2 of acetyl coenzyme A carboxylase
UGT1A1	UDP-glucuronosyltransferases
VRC07	Anti-HIV monoclonal antibody
αGLA	α-galactosidase A
αSG	Alpha-sarcoglycan
γ-sarcoglycan	Sarcoglycan gamma

Supplementary Table 5 | Background and progression of AAV clinical trials (duplicates excluded)
(*NIH = National Institutes of Health)

NCT Number	Funded By	Phases	Completion date
NCT00004533	*NIH	Phase 1	August, 2002
NCT00195143	Industry	Phase 1	August, 2005
NCT00617032	Industry	Phase 1	November, 2005
NCT00482027	Industry	Phase 1	January, 2007
NCT00252850	Industry	Phase 1	March, 2007
NCT00888446	Industry	Phase 2	December, 2007
NCT00400634	Industry	Phase 2	November, 2008
NCT00126724	Industry	Phase 1 / 2	May, 2009
NCT00087789	Industry	Phase 1	May, 2010
NCT01344798	Unknown	Phase 1	June, 2010
NCT00428935	Industry	Phase 1	July, 2010
NCT00891306	Industry	Phase 2 / 3	April, 2011
NCT00494195	NIH	Phase 1	August, 2011
NCT00454818	Industry	Phase 1 / 2	August, 2012
NCT00229736	Industry	Phase 1	March, 2013
NCT01109498	Industry	Phase 2 / 3	June, 2013
NCT02082860	Industry	Phase 1	November, 2014
NCT00643747	Industry	Phase 1 / 2	December, 2014
NCT00430768	NIH|Industry	Phase 1	January, 2015
NCT00534703	Industry	Phase 2	September, 2015
NCT01054339	Industry|NIH	Phase 2	October, 2015
NCT00976352	NIH	Phase 1 / 2	December, 2015
NCT01966887	Industry	Phase 2	February, 2016
NCT01643330	Industry	Phase 2	February, 2016
NCT01620801	Industry	Phase 1	March, 2016
NCT01899092	Industry	Phase 1 / 2	November, 2016
NCT02053064	Industry	Phase 1 / 2	June, 2017
NCT01494805	Industry	Phase 1 / 2	August, 2017
NCT01519349	Unknown	Phase 1	October, 2017
NCT01461213	Unknown	Phase 1 / 2	October, 2017
NCT02354781	Unknown	Phase 1 / 2	November, 2017
NCT02122952	Industry	Phase 1	December, 2017
NCT01414985	Unknown	Phase 1 / 2	October, 2017
NCT00985517	Industry	Phase 1 / 2	February, 2017
NCT02618915	Industry	Phase 1 / 2	June, 2017
NCT00749957	Industry	Phase 1 / 2	January, 2017
NCT02553135	Unknown	Phase 2	February, 2018
NCT02671539	Unknown	Phase 2	February, 2018
NCT01937455	NIH	Phase 1	February, 2018
NCT01024998	Industry	Phase 1	July, 2018
NCT02781480	Industry	Phase 1 / 2	December, 2018
NCT02652780	Industry	Phase 3	December, 2018
NCT00516477	Industry	Phase 1	July, 2018

NCT02418598	Industry	Phase 1 / 2	August, 2018
NCT00151216	Unknown	Phase 1	June, 2019
NCT02710500	Unknown	Phase 1	July, 2019
NCT02484092	Industry	Phase 2	July, 2019
NCT02652767	Industry	Phase 3	August, 2019
NCT03306277	Industry	Phase 3	January, 2019
NCT03300453	Industry	Phase 1 / 2	April, 2019
NCT03001310	Industry	Phase 1 / 2	August, 2019
NCT00377416	NIH	Early Phase 1	January, 2020
NCT03445715	Industry	Phase 1	January, 2020
NCT03252847	Industry	Phase 1 / 2	November, 2020
NCT04110964	Industry	Phase 1	December, 2020
NCT03496012	Unknown	Phase 3	October, 2020
NCT01395641	Unknown	Phase 1 / 2	January, 2020
NCT01161576	NIH	Phase 1	February, 2020
NCT01973543	Industry	Phase 1	December, 2020
NCT03116113	Unknown	Phase 1 / 2	March, 2020
NCT02651675	Industry|NIH	Phase 1 / 2	May, 2020
NCT03461289	Industry	Phase 3	November, 2020
NCT04133649	Industry	Phase 1	January, 2021
NCT04133454	Industry	Phase 1	January, 2021
NCT02695160	Industry	Phase 1	April, 2021
NCT03066258	Industry	Phase 1 / 2	May, 2021
NCT02852213	NIH	Phase 1	June, 2021
NCT02240407	Industry	Phase 1	June, 2021
NCT02407678	Unknown	Phase 2	August, 2021
NCT04135300	Unknown	N/A	November, 2021
NCT02725580	Industry	Phase 1 / 2	November, 2021
NCT03517085	Industry	Phase 1 / 2	December, 2021
NCT03065192	Industry	Phase 1	December, 2021
NCT02991144	Industry	Phase 1 / 2	December, 2021
NCT02396342	Industry	Phase 1 / 2	April, 2021
NCT01687608	Industry	Phase 1 / 2	August, 2021
NCT04728841	Unknown	N/A	March, 2021
NCT03223194	Industry	Phase 1 / 2	July, 2021
NCT03505099	Industry	Phase 3	May, 2021
NCT03369444	Unknown	Phase 1	November, 2021
NCT03381729	Industry	Phase 1	December, 2021
NCT03837184	Industry	Phase 3	February, 2021
NCT03758404	Industry	Phase 1 / 2	January, 2022
NCT02926066	Unknown	Phase 2	January, 2022
NCT04418427	Industry	Phase 2	January, 2022
NCT03003533	Industry	Phase 1 / 2	March, 2022
NCT03612869	Industry	Phase 2 / 3	March, 2022
NCT04567550	Industry	Phase 2	May, 2022
NCT04043104	Industry	Phase 1	May, 2022
NCT02702115	Industry	Phase 1 / 2	June, 2022

NCT03041324	Industry	Phase 1 / 2	June, 2022
NCT03748784	Industry	Phase 1	June, 2022
NCT03406104	Industry	Phase 3	August, 2022
NCT02727764	Industry	Phase 1	September, 2022
NCT02341807	Industry	Phase 1 / 2	October, 2022
NCT03734588	Industry	Phase 1 / 2	December, 2022
NCT04040049	Industry	Phase 1 / 2	December, 2022
NCT02790723	Unknown	Phase 1	December, 2022
NCT03562494	Industry	Phase 2	December, 2022
NCT01621581	NIH	Phase 1	May, 2022
NCT04514653	Industry	Phase 2	August, 2022
NCT03507686	Unknown	Phase 2	September, 2022
NCT03587116	Industry	Phase 3	January, 2022
NCT04671433	Industry	Phase 3	September, 2022
NCT03173521	Unknown	Phase 1 / 2	January, 2022
NCT02446249	Industry|NIH	Phase 1	November, 2022
NCT02161380	NIH	Phase 1	March, 2023
NCT04693598	Industry	Phase 1 / 2	April, 2023
NCT04483440	Industry	Phase 1	May, 2023
NCT03533673	Industry|NIH	Phase 1 / 2	June, 2023
NCT01482195	Unknown	Phase 1	August, 2023
NCT03770572	Industry	Phase 1 / 2	September, 2023
NCT04517149	Industry	Phase 1 / 2	September, 2023
NCT04093349	Industry	Phase 1 / 2	October, 2023
NCT02416622	Industry	Phase 1 / 2	October, 2023
NCT04088734	Industry	Phase 1 / 2	December, 2023
NCT03634007	Industry	Phase 1	February, 2023
NCT03520751	Unknown	Phase 1 / 2	March, 2023
NCT03489291	Industry	Phase 2	December, 2023
NCT04046224	Industry	Phase 1 / 2	February, 2024
NCT04704921	Industry	Phase 2 / 3	March, 2024
NCT02576795	Industry	Phase 1 / 2	March, 2024
NCT03199469	Industry	Phase 1 / 2	March, 2024
NCT04680065	Industry	Phase 1	April, 2024
NCT04783181	Industry	Phase 1 / 2	December, 2024
NCT03278873	Industry	Phase 1 / 2	September, 2024
NCT03061201	Industry	Phase 2	April, 2024
NCT03293524	Industry	Phase 3	March, 2024
NCT04394286	Industry	Phase 1 / 2	June, 2024
NCT04833907	Industry	Phase 1 / 2	September, 2024
NCT03882437	Industry	Phase 1	July, 2024
NCT03846193	Industry	Phase 1 / 2	February, 2025
NCT03569891	Industry	Phase 3	March, 2025
NCT04273269	Industry	Phase 1 / 2	June, 2025
NCT03001830	Unknown	Phase 1	June, 2025
NCT02935517	Industry	Phase 1 / 2	September, 2025
NCT02599922	Industry|NIH	Phase 1 / 2	September, 2025

NCT03326336	Industry	Phase 1 / 2	December, 2025
NCT04611503	Unknown	Phase 1 / 2	December, 2025
NCT04240314	Industry	Phase 1 / 2	November, 2025
NCT02317887	NIH	Phase 1 / 2	July, 2025
NCT02077361	Unknown	Phase 1 / 2	October, 2025
NCT04676048	Industry	Phase 1 / 2	April, 2026
NCT04120493	Industry	Phase 1 / 2	May, 2026
NCT04167540	Industry	Phase 1	June, 2026
NCT03316560	Industry	Phase 1 / 2	August, 2026
NCT03328130	Industry	Phase 1 / 2	September, 2026
NCT03520712	Industry	Phase 1 / 2	November, 2026
NCT04455230	Industry	N/A	December, 2026
NCT03466463	Unknown	N/A	May, 2026
NCT03952637	NIH	Phase 1 / 2	June, 2026
NCT03588299	Industry	Phase 1 / 2	November, 2026
NCT03362502	Industry	Phase 1	June, 2026
NCT04516369	Industry	Phase 3	October, 2026
NCT04370054	Industry	Phase 3	September, 2026
NCT03370172	Industry	Phase 1 / 2	February, 2026
NCT04174105	Industry	Phase 1 / 2	January, 2027
NCT03368742	Industry	Phase 1 / 2	April, 2027
NCT04519749	Industry	Phase 1 / 2	April, 2027
NCT04684940	Industry	Phase 1 / 2	June, 2027
NCT04127578	Industry	Phase 1 / 2	June, 2027
NCT04537377	Industry	Phase 1 / 2	July, 2027
NCT04747431	Industry	Phase 1 / 2	August, 2027
NCT04408625	Industry	Phase 1 / 2	December, 2027
NCT04480567	Industry	Phase 1 / 2	December, 2027
NCT04794101	Industry	Phase 3	October, 2027
NCT03374202	NIH	Phase 1	April, 2027
NCT04798235	Unknown	Phase 1 / 2	May, 2027
NCT03307980	Industry	Phase 2	December, 2027
NCT04411654	Industry	Phase 1 / 2	April, 2028
NCT04669535	Industry	Phase 1	June, 2028
NCT04703842	Industry	Phase 1 / 2	December, 2028
NCT04850118	Industry	Phase 2 / 3	March, 2029
NCT00999609	Industry	Phase 3	July, 2029
NCT04737460	Unknown	Phase 1	June, 2029
NCT01208389	Industry	Phase 1 / 2	June, 2030
NCT00979238	NIH	Phase 1	April, 2032
NCT00076557	Industry	Phase 1 / 2	N/A
NCT02346422	Industry	Phase 1 / 2	N/A
NCT00643890	Industry	Phase 2	N/A

References (Supplementary Table 2)

1. Naso MF, Tomkowicz B, Perry WL, 3rd, Strohl WR. Adeno-Associated Virus (AAV) as a Vector for Gene Therapy. BioDrugs : clinical immunotherapeutics, biopharmaceuticals and gene therapy. 2017;31(4):317-34. doi: 10.1007/s40259-017-0234-5 [doi].
2. Hocquemiller M, Giersch L, Audrain M, Parker S, Cartier N. Adeno-Associated Virus-Based Gene Therapy for CNS Diseases. Human Gene Therapy. 2016;27(7):478-96. doi: 10.1089/hum.2016.087 [doi].
3. Mendell JR, Sahenk Z, Lehman K, Nease C, Lowes LP, Miller NF, et al. Assessment of Systemic Delivery of rAAVrh74.MHCK7.micro-dystrophin in Children With Duchenne Muscular Dystrophy: A Nonrandomized Controlled Trial. JAMA neurology. 2020;77(9):1122-31. doi: 10.1001/jamaneurol.2020.1484.
4. Thwaite R, Pages G, Chillon M, Bosch A. AAVrh.10 immunogenicity in mice and humans. Relevance of antibody cross-reactivity in human gene therapy. Gene Ther. 2015;22(2):196-201. Epub 2014/11/21. doi: 10.1038/gt.2014.103. PubMed PMID: 25410741.
5. Pipe S, Becka M, Detering E, Vanevski K, Lissitchkov T. First-in-human Gene Therapy Study of AAVhu37 Capsid Vector Technology in Severe Hemophilia A. Blood. 2019;134(Supplement_1):4630-. doi: 10.1182/blood-2019-125764.
6. Petrs-Silva H, Dinculescu A, Li Q, Min SH, Chiodo V, Pang JJ, et al. High-efficiency transduction of the mouse retina by tyrosine-mutant AAV serotype vectors. Molecular therapy : the journal of the American Society of Gene Therapy. 2009;17(3):463-71. doi: 10.1038/mt.2008.269 [doi].
7. Bennett A, Keravala A, Makal V, Kurian J, Belbellaa B, Aeran R, et al. Structure comparison of the chimeric AAV2.7m8 vector with parental AAV2. Journal of structural biology. 2020;209(2):107433. doi: https://doi.org/10.1016/j.jsb.2019.107433.
8. Butterfield JSS, Hege KM, Herzog RW, Kaczmarek R. A Molecular Revolution in the Treatment of Hemophilia. Molecular Therapy. 2020;28(4):997-1015. doi: https://doi.org/10.1016/j.ymthe.2019.11.006.
9. Powell SK, Khan N, Parker CL, Samulski RJ, Matsushima G, Gray SJ, et al. Characterization of a novel adeno-associated viral vector with preferential oligodendrocyte tropism. Gene therapy. 2016;23(11):807-14. doi: 10.1038/gt.2016.62 [doi].
10. El Andari J, Grimm D. Production, Processing, and Characterization of Synthetic AAV Gene Therapy Vectors. Biotechnology Journal. 2021;16(1):2000025. doi: https://doi.org/10.1002/biot.202000025.
11. Lisowski L, Dane AP, Chu K, Zhang Y, Cunningham SC, Wilson EM, et al. Selection and evaluation of clinically relevant AAV variants in a xenograft liver model. Nature. 2014;506(7488):382-6. doi: 10.1038/nature12875.
12. Perrin GQ, Herzog RW, Markusic DM. Update on clinical gene therapy for hemophilia. Blood. 2019;133(5):407-14. doi: 10.1182/blood-2018-07-820720.
13. Whittlesey K, Brooks G, Croze R, Schmitt C, Szymanski P, Nye J, et al. A novel cardiotropic AAV variant 4D-C102 demonstrates: superior gene delivery and reduced immunogenicity in cardiac tissues versus wildtype AAV in non-human primates, and results in functional GLA in cardiomyocytes and Fabry fibroblasts. 2019;(4D Molecular Therapeutics).
14. Therapeutics DM. Annual Report. United States Securities and Exchange Commission, 2019 Contract No.: Report.
