# Supplementary figures and images for: Gene Therapy Advances: A Meta-Analysis of AAV Usage in Clinical Settings
Source: Front Med (Lausanne). 2022 Feb 9;8:809118. doi: 10.3389/fmed.2021.809118 (PMC8864161; doi:10.3389/fmed.2021.809118)

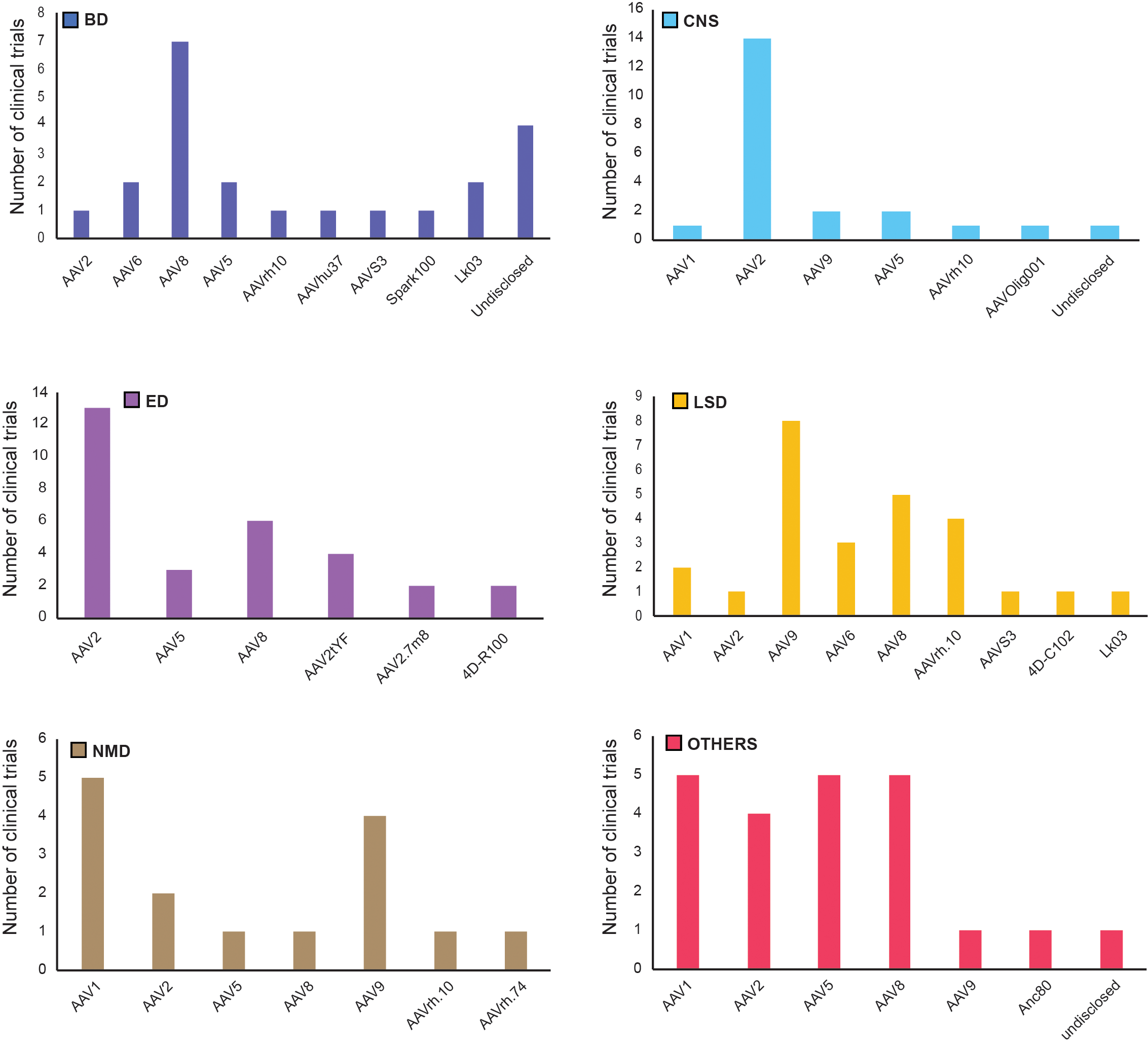

Supplement: Supplementary file 3 [file Image_1.TIF]

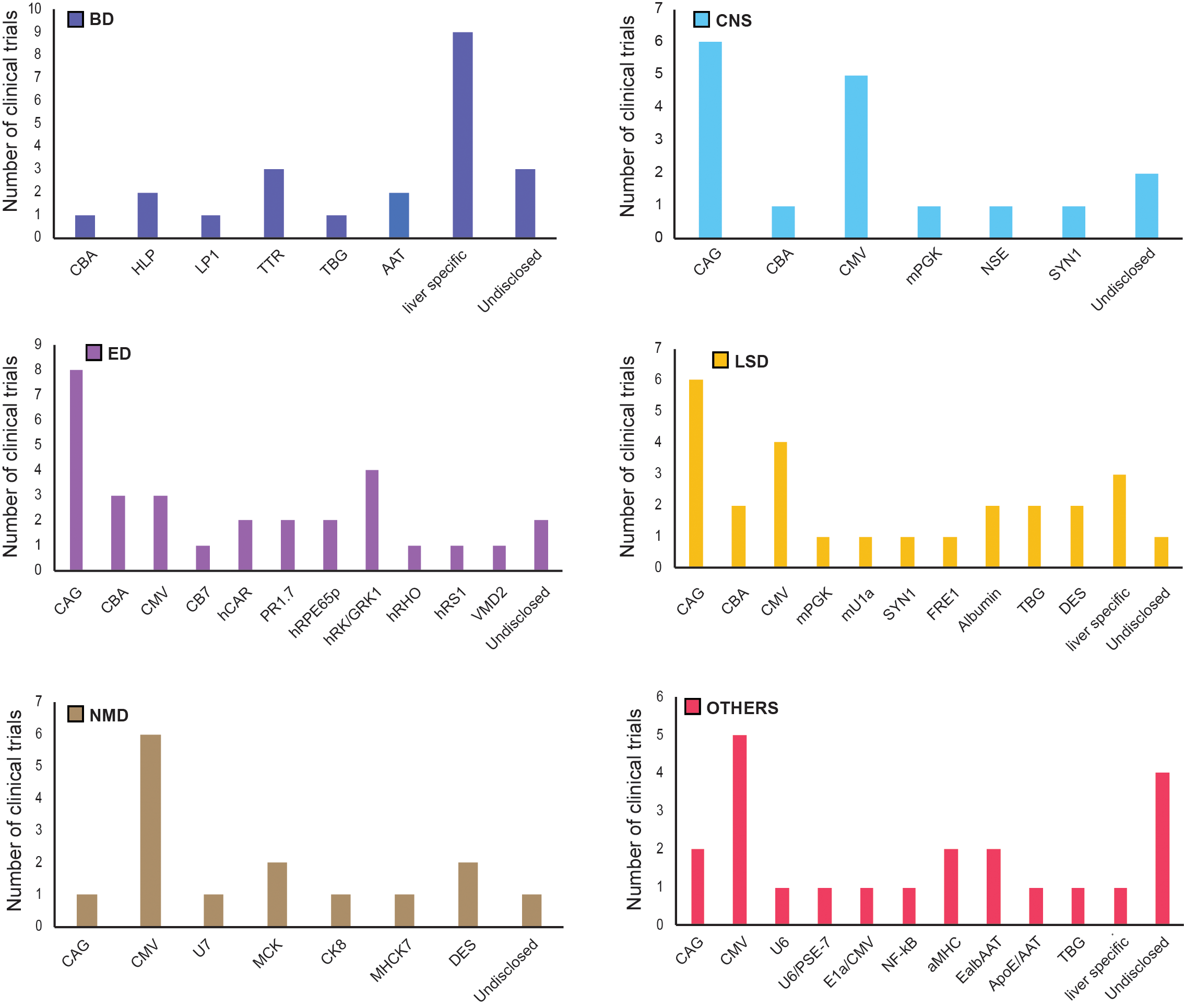

Supplement: Supplementary file 4 [file Image_2.TIF]
